# Difluoromethyl-1,3,4-oxadiazoles are slow-binding substrate analog inhibitors of histone deacetylase 6 with unprecedented isotype selectivity

**DOI:** 10.1016/j.jbc.2022.102800

**Published:** 2022-12-15

**Authors:** Edoardo Cellupica, Gianluca Caprini, Paola Cordella, Cyprian Cukier, Gianluca Fossati, Mattia Marchini, Ilaria Rocchio, Giovanni Sandrone, Maria Antonietta Vanoni, Barbara Vergani, Karol Źrubek, Andrea Stevenazzi, Christian Steinkühler

**Affiliations:** 1 Research and Development, Italfarmaco Group, Cinisello Balsamo, Italy; 2 Department of Biochemistry, Selvita S.A., Kraków, Poland; 3 Department of Biosciences, University of Milan, Milan, Italy

**Keywords:** histone deacetylase, histone deacetylase inhibitor, histone deacetylase 6, inhibition mechanism, epigenetics, X-ray crystallography, difluoromethyl oxadiazole, zinc-binding group, mechanism-based inhibitor, CD, catalytic domain, DFMO, difluoromethyl-1,3,4-oxadiazole, HB, hydrogen bond, HDACi, HDAC inhibitor, HDAC6, histone deacetylase 6, PDB, Protein Data Bank, QM/MM, quantum mechanical/molecular mechanical, ZBG, Zn-binding group, Zn, zinc

## Abstract

Histone deacetylase 6 (HDAC6) is an attractive drug development target because of its role in the immune response, neuropathy, and cancer. Knockout mice develop normally and have no apparent phenotype, suggesting that selective inhibitors should have an excellent therapeutic window. Unfortunately, current HDAC6 inhibitors have only moderate selectivity and may inhibit other HDAC subtypes at high concentrations, potentially leading to side effects. Recently, substituted oxadiazoles have attracted attention as a promising novel HDAC inhibitor chemotype, but their mechanism of action is unknown. Here, we show that compounds containing a difluoromethyl-1,3,4-oxadiazole (DFMO) moiety are potent and single-digit nanomolar inhibitors with an unprecedented greater than 10^4^-fold selectivity for HDAC6 over all other HDAC subtypes. By combining kinetics, X-ray crystallography, and mass spectrometry, we found that DFMO derivatives are slow-binding substrate analogs of HDAC6 that undergo an enzyme-catalyzed ring opening reaction, forming a tight and long-lived enzyme–inhibitor complex. The elucidation of the mechanism of action of DFMO derivatives paves the way for the rational design of highly selective inhibitors of HDAC6 and possibly of other HDAC subtypes as well with potentially important therapeutic implications.

Zinc (Zn)-dependent histone deacetylases (HDACs) are a family of 11 evolutionarily related hydrolases that catalyze the removal of acyl residues from histones, non-histone proteins, and polyamines (1, 2, 3). Given the involvement of HDACs in numerous diseases, the pharmaceutical industry is pursuing the development of HDAC inhibitors (HDACis) since almost 2 decades, an effort that led to the approval of five molecules for the treatment of cancer (4). Unfortunately, the therapeutic benefit of HDACis has been limited by side effects because of the poor selectivity of these first-generation molecules, which inhibit several to all the Zn-dependent HDAC family members, affecting crucial physiological functions.

HDAC6 stands out among the 11 human isoforms, as being the only one that has two homologous tandem catalytic domains (CD1 and CD2). Furthermore, HDAC6 is mainly localized in the cytoplasm, and its main substrates are various non-histone proteins, such as α-tubulin, Foxp3, Hsp90, β-catenin, cortactin, and peroxiredoxins. HDAC6 knockout mice are viable and fertile and do not show overt physiological dysfunctions (5). Also, selective HDAC6 inhibition was demonstrated to be well tolerated in both preclinical species and in human clinical trials (6).

Since HDAC6 plays a role in regulating immune response, in the development of neuropathies and in Alzheimer’s disease, the identification of highly specific HDAC6is is of great importance (7).

The classical HDACi pharmacophore consists of a Zn-binding group (ZBG), interacting with the active site Zn ion, a cap, which interacts with the outer region of the enzyme, and a linker that connects the ZBG to the cap. Most HDACis have a hydroxamic acid moiety as ZBG, a chemical group that has inherent stability and safety issues. Recently, reports on oxadiazole-based HDAC6is (Fig. S1) as a potential alternative to hydroxamates appeared, but their mechanism of action is unclear (8).

Focusing on difluoromethyl-1,3,4-oxiadiazole (DFMO)-containing HDAC6is, we have been able to identify molecules with unprecedented selectivity for HDAC6 over all other HDACs and excellent drug-like properties (9). Using X-ray crystallography, molecular modeling, kinetics, and rapid chromatography coupled to mass spectrometry, we show that DMFO-containing HDACis are actually substrate analogs of HDAC6 that undergo an enzyme-catalyzed ring hydration leading to a tight and long-lived enzyme–inhibitor complex. Our findings open the way for the development of novel HDAC6 selective inhibitors for the treatment of neuropathies and other diseases, but they also point to the intriguing possibility to use oxadiazoles in the development of selective inhibitors of other HDAC subtypes.

## Results and discussion

### Compound **1** is a potent and CD2-selective slow binding inhibitor of HDAC6

In our medicinal chemistry program, we identified potent and selective HDAC6is in the DFMO series using *in silico* modeling and traditional structure–activity relationship (article in preparation). The DFMO containing inhibitor **1** (Fig. S1) (9), which was selected as an example for this communication, is at least four orders of magnitude more potent on HDAC6 than on any of the other HDACs (Table 1). This selectivity is remarkable when compared with hydroxamate-based HDAC6is like ACY-1215 (Ricolinostat), ACY-241 (Citarinostat), KA2507 (10), and ITF3756 (11) (Fig. S2 and Table S1). Furthermore, we carried out inhibition measurements to investigate the interdomain selectivity of **1** using the isolated CD1 (zHDAC6-CD1) and CD2 (zHDAC6-CD2) domains of zebrafish HDAC6. In agreement with previous works (12, 13), when tested on commercial fluorogenic substrate Fluor De Lys Green, zHDAC6-CD1 exhibited a weaker but measurable activity compared with that of zHDAC6-CD2 (data not shown). More interestingly, we found that **1** is highly selective for the CD2 domain (Tables 1 and S1). Our data confirmed that the deacetylase activity of the full-length HDAC6 on commercial fluorogenic substrate Fluor De Lys Green mainly resides in CD2 and that HDAC6is selectively target CD2 for their activity. The only crystal structure of HDAC6 CD1 complexed with an HDAC6i has recently been reported (12). In this context, the unique K330 in CD1 has been reported to interact with bound inhibitors (12, 14) and to confer specificity for the deacetylation of peptide substrates bearing C-terminal acetyl-lysine residues (13, 14, 15). As with substrate preferences, binding of **1** and hydroxamate-based HDAC6is to the CD1 active site could be disfavored by suboptimal interactions with this key residue.

**Table 1:** Inhibitory profile of **1**, **2**, and **3** on HDACs

HDAC class				
		Inhibitors		
	R			
	IC50 (μM)			
Class I	HDAC1	>100	>100	14.7 ± 0.4
	HDAC2	>100	>100	43.6 ± 1.8
	HDAC3	>100	46.8 ± 5.0	2.01 ± 0.09
	HDAC8	>100	>100	10.2 ± 0.3
Class IIb	HDAC6	0.0077 ± 0.0003	1.58 ± 0.05	0.329 ± 0.006
	zHDAC6-CD2	0.0078 ± 0.0004	2.25 ± 0.04	0.097 ± 0.004
	zHDAC6-CD1	82.2 ± 7.7	>100	13.9 ± 0.6
	HDAC10	>100	>100	13.30 ± 0.06
Class IIa	HDAC4	>100	>100	>100
	HDAC5	>100	>100	98.5 ± 7.2
	HDAC7	>100	>100	64.2 ± 2.4
	HDAC9	>100	>100	46.4 ± 0.9
Class IV	HDAC11	>100	>100	>100

In the presence of **1**, we noticed that inhibition increases during the time course of the assay, suggesting slow binding inhibition (Fig. S3*A*). Slow binding inhibition may result from various mechanisms (16). The most common ones may be discerned by determining the dependence of the observed pseudo–first-order rate constant *k*_obs_ of formation of the enzyme–inhibitor complex on the inhibitor concentration. We ruled out that the inhibitor itself underwent hydration–dehydration equilibria in aqueous solution with only one species binding to the enzyme (17), as we were unable to detect a hydrated form of **1** in solution. Thus, the observed linear dependence of *k*_obs_ on inhibitor concentration is consistent with the binding process itself being slow, with an apparent association rate constant of 9.6 × 10^5^ M^−1^ min^−1^ (Fig. S3, *A* and *B* and Table 2). The magnitude of the apparent dissociation rate constant was calculated from the *k*_obs_ plot and jump dilution assays (Table 2 and Fig. S3, *B* and *C*) yielding similar values (4.6 ± 2.3 [×10^−3^] min^−1^ *versus* 3.3 ± 0.2 [×10^−3^] min^−1^). The calculated residence time of the inhibitor was 4.5 to 5 h.

**Table 2:** Kinetic parameters of the inhibition of HDAC6 by **1**

HDAC6 forms	Parameters	**1**
Human HDAC6	IC_50_, nM	7.7 ± 0.3
		8.1 ± 0.3^a^
	*k*_on_^app^, M^−1^ min^−1^ (×10^5^)^b^	9.6 ± 0.3
		9.1 ± 0.3^a^
	*k*_off_^app^, min^−1^ (×10^−3^)^b^	4.6 ± 2.3
		6.7 ± 1.9^a^
	*k*_off_^app^, min^−1^ (×10^−3^)^c^	3.3 ± 0.2
	*K*_i_^app^, nM^d^	4.8 ± 2.4
		6.9 ± 1.9^a^
	Mode of inhibition	Slow-on/slow-off
zHDAC6-CD2 WT	IC_50_, nM	7.8 ± 0.4
	*k*_on_^app^, M^−1^ min^−1^ (×10^5^)^b^	15.1 ± 0.1
	*k*_off_^app^, min^−1^ (×10^−3^)^b^	12.8 ± 1.1
	*k*_off_^app^, min^−1^ (×10^−3^)^c^	9.6 ± 1.1
	*K*_i_^app^, nM^d^	8.5 ± 0.7
	Mode of inhibition	Slow-on/slow-off
zHDAC6-CD2 Y745F	IC_50_, nM	15.1 ± 0.9
	*k*_on_^app^, M^−1^ min^−1^ (×10^5^)^b^	∼10^10^^d^
	*k*_off_^app^, min^−1^ (×10^−3^)^c^	92.7 ± 3.2
	Mode of inhibition	Fast-on/slow-off

a Parameters determined in D_2_O.

b Calculated from *k*_obs_ plot, *K*_i_^app^ = *k*_off_^app^/*k*_on_^app^.

c *k*_off_^app^ values from jump-dilution assay.

d Parameter estimated from *k*_off_^app^/*K*_i_^app^ ratio.

The same slow-binding behavior was shown by **1** with both human full-length HDAC6 and zHDAC6-CD2 (Fig. S3). Moreover, the calculated IC_50_ values and kinetic constants are similar for both enzymes (Tables 2 and S3), confirming that zHDAC6-CD2 is a valid surrogate of human CD2 (12, 13, 18).

### X-ray crystal structure of the compound **1**–zHDAC6-CD2 complex

**Figure 1:**
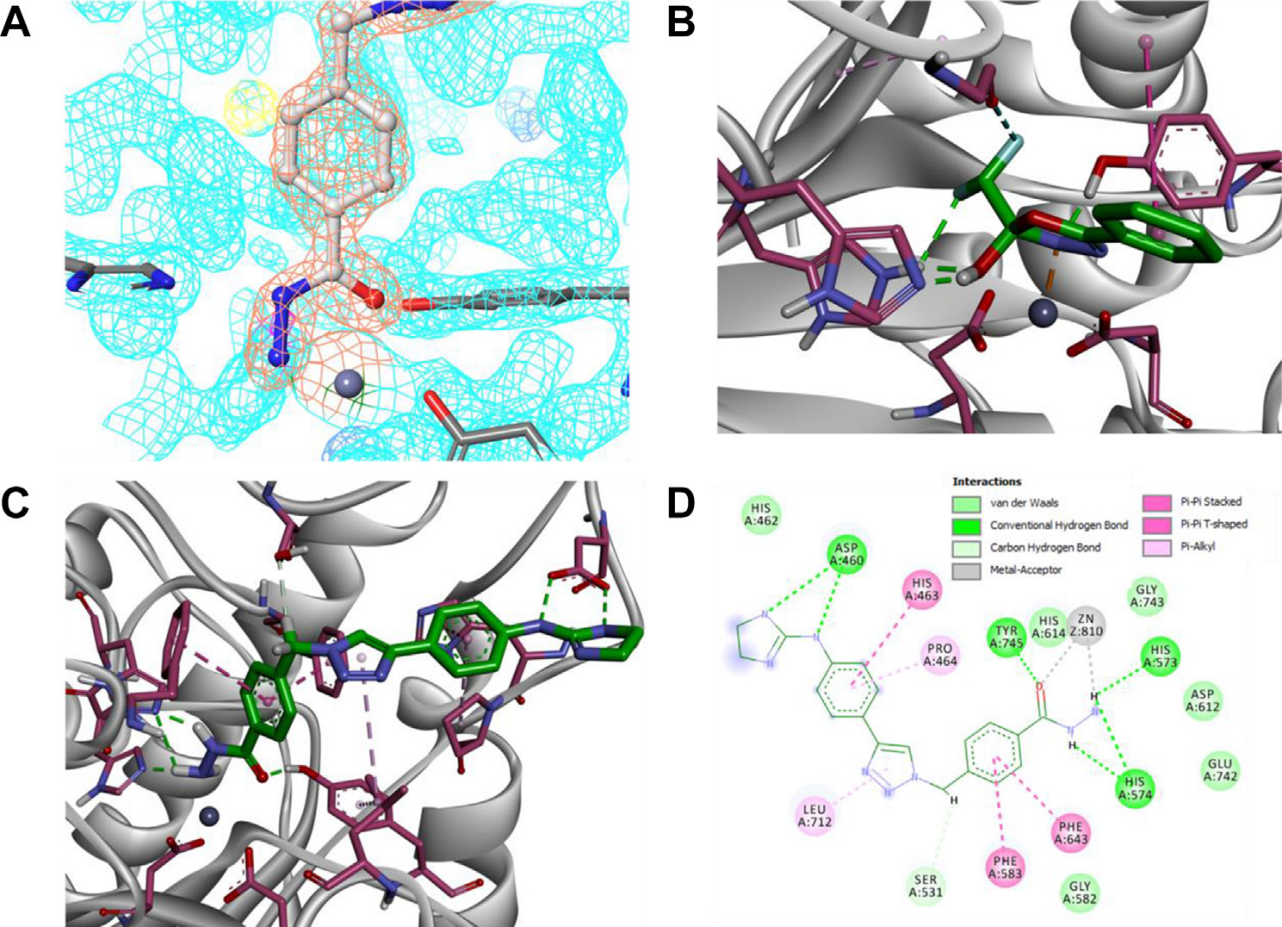
**Structural analysis.***A,* experimental electron density of ligand–zHDAC6-CD2 complex with hypothesized hydrazide-based analog. *B,* QM/MM optimized geometry of the hypothetical hydrate DFMO in active site. *C,* X-ray crystal structure of **1** bound to zHDAC6-CD2 showing the product hydrazide **3** in the catalytic core. *D,* bidimensional projection of binding site showing noncovalent interaction network between ligand and closest residues. DFMO, difluoromethyl-1,3,4-oxadiazole; HDAC6, histone deacetylase 6; QM/MM, quantum mechanical/molecular mechanical.

To gain further experimental insight into the mechanism of inhibition of HDAC6 by DFMOs, **1** was cocrystallized with zHDAC6-CD2. Superposition of the resulting complex (1.60 Å resolution; Protein Data Bank [PDB] code: 8A8Z) with the ligand-free protein (PDB code: 5EEM (13)) shows a root mean square deviation value of 0.378 Å, indicating that no major conformational changes were induced by binding of the inhibitor (crystallographic details in Table S2). Surprisingly, the refined electron density of the bound ligand was not compatible with the inhibitor structure and suggested that a DFMO ring opening reaction had occurred. Several reaction mechanisms were hypothesized, and the resulting substructures were aligned with the experimental electron density map. In this procedure, a hydrazide moiety showed the best fit to the experimental density (Fig. 1, *A* and *C*).

The postulated hydrazide could be formed as the result of two subsequent reactions. After the initial binding of the inhibitor, the Zn cation could enhance the electrophilic behavior of the oxadiazole sp^2^ carbon atom, allowing a nucleophilic attack by the catalytic water molecule. Following the proposed catalytic mechanism of HDAC6 (13), the solvent molecule could be activated for a nucleophilic attack by His573. The hydrated DFMO intermediate resulting from such bond rearrangements was modeled (Fig. 1*B*), revealing that this moiety is able to engage in hydrogen bond (HB) formation with the histidine dyad (involving fluorine atom and the entering OH group), preserving the original interaction with Tyr745. The hydrated intermediate can further undergo a ring-opening reaction to yield an acyl-hydrazide. In order to explain the hydrazide detected in the crystal structure, we hypothesized an additional hydrolysis reaction, which could be either enzyme catalyzed or take place in solution upon release of the acyl-hydrazide from the enzyme active site. If this second hydrolysis reaction was indeed enzyme catalyzed, the Zn coordination sphere would need to be restored by the entry of a second water molecule. The metal cation could then activate the difluoro-acyl carbonyl, allowing for the canonical deacetylation and the subsequent release of hydrazide and difluoroacetic acid.

In the crystal structure (Fig. 1, *C* and *D*), the hydrazide NH_2_ group is positioned to share a sp^3^ lone pair with the metal cation, whereas simultaneously acting as a HB donor with both catalytic key residues His573 and His574. The position of the ZBG carbonyl oxygen atom suggests a HB with Tyr745 rather than a bond with the Zn ion, indicating hydrazide monodenticity. The phenyl substructure directly bound to the ZBG is buried in the crevice made by Phe583 and Phe643, where aromatic rings are interacting through a three-body π–π interaction (19), whereas the C-H⋯X HB is formed with the methylene substructure and the key residue Ser531 (12, 13, 18). A weak π-alkyl (20) bond is established between triazole and Leu712, whereas the phenyl substructure of the cap interacts with His462 and Pro464. Finally, the apical 2-aminoimidazoline is bound to Asp460 through multiple HBs. Despite the large number of residues involved in α-tubulin recognition (12), the inhibitor apparently interacts only with this negatively charged amino acid, suggesting possible further optimizations involving moieties able to interact with W459 and/or with the α-helix facing the L1 loop.

### Molecular modeling

A conformation analysis of the inhibitor was done to cast light on the electronic properties of the oxadiazole ring. Density functional theory (B3LYP method, 6–311G∗∗++ basis set) global minimum refinement confirmed partial negative charges (natural bond orbitals) on aromatic nitrogen and oxygen atoms, whereas atomic Fukui indices indicated that the aromatic carbon atom bound to the CHF_2_ moiety exhibits a clear electrophilic behavior.

We next docked **1** into zHDAC6-CD2 (PDB code: 7O2R) using the Glide34 module (21) of Schrödinger. The final best pose indicated that the DFMO moiety can bind the Zn cation through the nitrogen close to the CHF_2_ group (Zn-N distance 2.4 Å), thereby suggesting a conventional dative bond between the lone pair donor and the Zn ion. The DFMO group, even with its relatively large volume, can fit into the catalytic pocket, placing the difluoromethyl moiety in an inner crevice delimited by Pro571, Cys584, Gly582, and the catalytic dyad His573-His574. In this pose, the inhibitor undergoes multiple noncovalent interactions between halogen atoms and Gly582 and Cys584 as well as π–π stacking interactions between the 1,3,4-oxadiazole and the His573-His574 dyad. Interestingly, the area delimited by the DFMO group, the Zn cation, His614, His573, and His574 defines a residual empty space (about 60 Å^3^) that is sufficiently broad to accommodate a water molecule. Additional docking of a single solvent molecule allows to detect a suitable pose with the water oxygen atom at a distance of 2.2 Å from the DFMO centroid. A supplementary refinement was done using the quantum mechanical/molecular mechanical (QM/MM) method (22). The optimized geometry indicates that the solvent molecule is in the Zn coordination sphere (d_Zn-O_ = 2.08 Å), hydrogen bonded to both catalytic histidines (d_N-H_ = 1.90 Å and d_N-H_ = 1.58 Å with His573 and His574, respectively), whereas the oxygen atom is oriented toward the electrophilic center of the oxadiazole, at a distance (d_O-C_ = 2.79 Å) compatible with a nucleophilic attack (Fig. S4*A*).

The possible simultaneous presence of a water molecule in the metal cation coordination sphere and of a ZBG that is bulkier than a hydroxamic acid was unexpected. In order to evaluate if this can be related to the high selectivity toward other HDACs, analogous modeling experiments were performed with HDAC1 (PDB code: 5ICN (23)), as a representative of class I. The optimized geometry obtained with the QM/MM method (Fig. S4*B*) shows a water molecule in the Zn coordination sphere (d_Zn-O_ = 2.12 Å), which is still able to engage in two HBs with the catalytic histidine dyad (*e.g.*, His140 and His141). However, the water oxygen atom is positioned closer to oxadiazole centroid (2.86 Å) than to aromatic electrophilic carbon atom (3.07 Å), which is exactly the opposite of what is observed in HDAC6 (3.31 and 2.79 Å, respectively). In addition to suboptimal bond angles, the lone pair of electrons not involved in the interaction with the metal ion occupies a position not suitable for nucleophilic attack, since this unshared electron pair is trapped in a n→π∗ interaction (24), involving an antibonding orbital mainly centered on the oxadiazole. The differences between HDAC subtypes can be explained in terms of a deeper penetration of the DFMO moiety in class I enzymes because of an inner empty space. On the contrary, residues R_569_P_570_P_571_ in the active site of HDAC6 prevent this deep entry and allow an optimal orientation for nucleophilic attack.

An intermediate behavior is detected when CD1 is involved in a complex with **1**. Docking indicates that both DFMO and additional water molecules can be accommodated in the catalytic pocket, whereas the QM/MM approach reveals that the phenyl–DFMO moiety penetrates deeper into the CD1 catalytic pocket, suggesting an unsuitable initial geometry for the first hydrolysis step (Fig. S4*C*). Interestingly, both features can be observed in the Nexturastat A–CD1 complex (PDB code: 5G0I) (12) (Fig. S4). Superposition of **1** with Nexturastat A enzyme–bound conformers further suggests a key role of L712, which contributes to form a flat hydrophobic pocket in CD2, able to accommodate relatively large aromatic moieties. This pocket is not formed in CD1 because of the presence of the protruding and flexible side chain of K330 (13), which is likely to interact unfavorably with aromatic cap terms, forcing inhibitors into a less favorable binding pose (Fig. S4*F*). Finally, it should be noticed that **1** could experience an additional nonbonding effect related to the repulsive electrostatic interaction between the lysine side chain and the positively charged dihydroimidazolamine moiety of both ligands. Moreover, the hydrophobic crevice (12) surrounding the phenyl substructure close to the DFMO moiety contains a tryptophan residue (W261) instead of a phenylalanine (F643), leading to a final geometry governed by a peculiar π–π interaction, probably able to bury the inhibitor deeper in the catalytic pocket.

### Two-step hydrolytic conversion of the DFMO inhibitor by HDAC6

To provide experimental confirmation of this mechanism, acyl-hydrazide **2** and hydrazide **3** (Table 1) were synthesized and used as standards for mass spectrometry studies. Unfortunately, because of its chemical instability, it was not possible to prepare the postulated closed hydrated intermediate, which is not distinguishable from **2** by mass spectrometry.

We characterized **2** and **3** in more detail: both compounds are far less potent but still quite selective HDAC6is, with **2** showing a higher selectivity than **3** (Table 1). Both compounds are fast-on and fast-off inhibitors of HDAC6 (Fig. S3, *G*–*N*) and all the other HDAC isoforms (data not shown).

**Figure 2:**
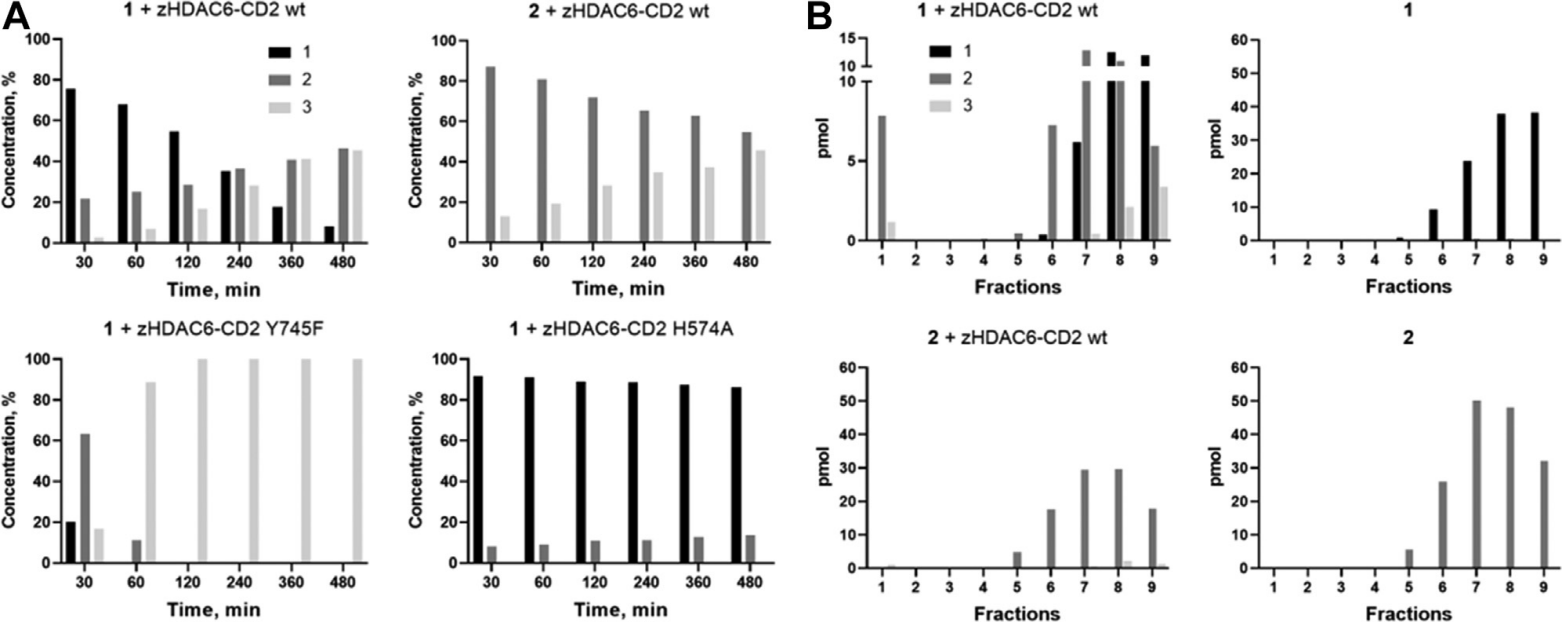
**Two-step hydrolytic conversion of 1.***A,* the time course of **1** and **2** (5 μM) consumption during incubation at 25 °C with zHDAC6-CD2 WT, Y745F, or H574A forms (1 μM) as detected by LC–HRMS analysis. Bars refer to the percentage of compound concentration at the indicated times. *B*, **1** and **2** were incubated with enzyme for 6 h at 25 °C, an aliquot was loaded on a spin column and the eluted fractions were analyzed by LC-HRMS for identification of the HDAC6-inhibitors complexes. HDAC6, histone deacetylase 6; HRMS, high-resolution mass spectrometry.

**Figure 3:**
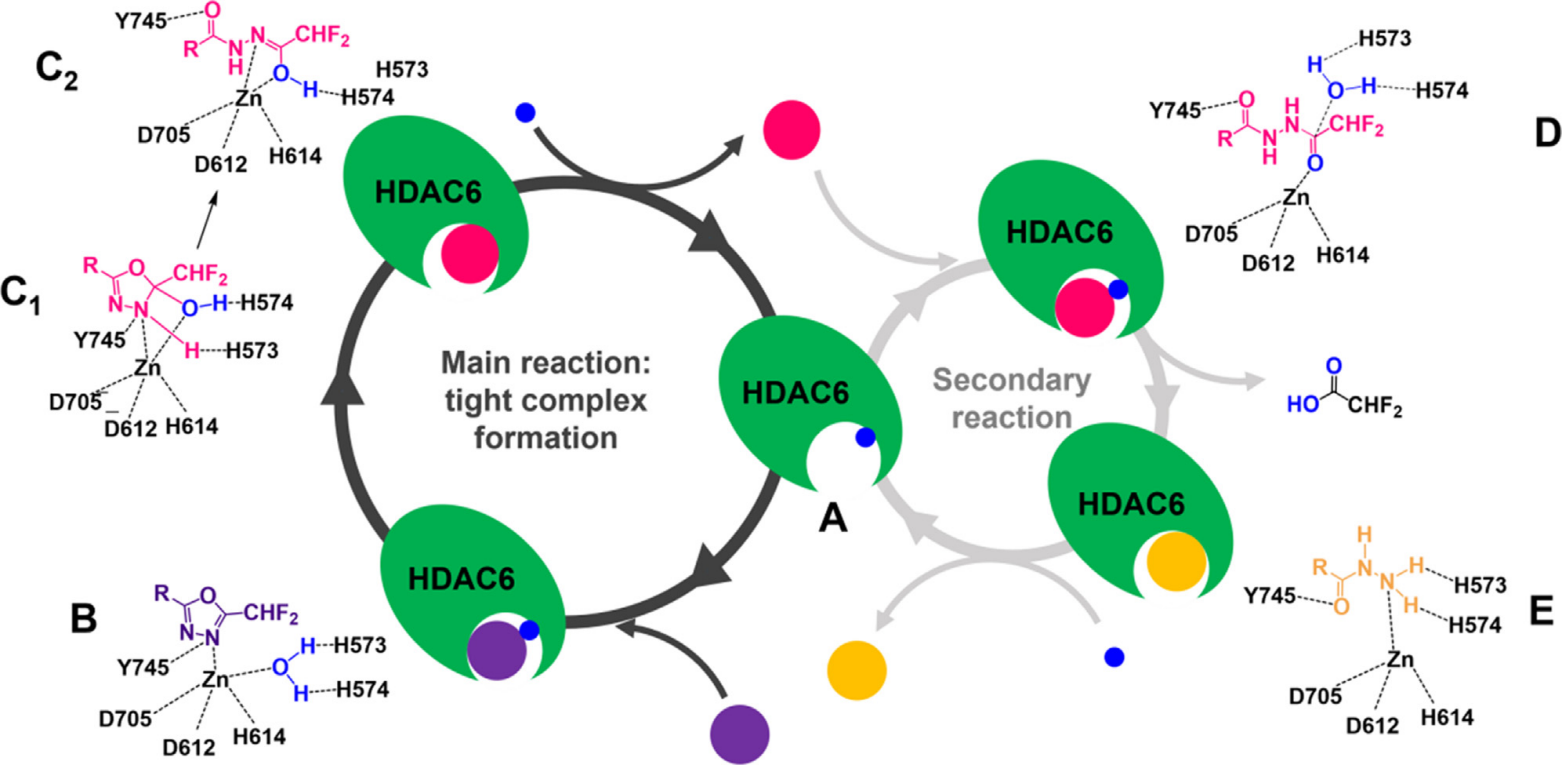
**Proposed reaction mechanism.***A,* free enzyme. *B,* binding of **1** to the enzyme active site with formation of the noncovalent complex. *C,* formation of the tight binding long-lived intermediate. Closed hydrated intermediate (C_1_) or acyl-hydrazide (C_2_) is not distinguishable. *D,* at high concentrations, **2** can rebind to enzyme and be hydrolyzed to **3** (*E*) and be released. Water, **1**, **2,** and **3** are colored in *blue*, *violet*, *pink*, and *yellow*, respectively.

When incubated with zHDAC6-CD2, **1** is converted into **2** first and **3** thereafter (Fig. 2*A*). The observed first-order rate constant for hydrolysis of **1** (6.04 ± 0.22 [×10^−3^] min^−1^) was similar to the apparent *k*_off_ value calculated by kinetic analysis (Table 2), suggesting that the slow dissociation of a hydrated derivative of **1** is actually measured. The linear dependence of the observed rate of onset of inhibition can therefore be ascribed to formation of an initial enzyme–inhibitor complex, which is characterized by a high dissociation constant, well above the range of concentrations used experimentally. Furthermore, **2** was converted into **3** by zHDAC6-CD2 (Fig. 2*A*). These experiments indicate that both **1** and **2** are substrates of HDAC6. However, we notice that formation of **3** requires high concentrations of **2** (Fig. 3), which are built up under crystallization conditions, but are unlikely to become relevant *in vivo*.

To identify the long-lived inhibitor–HDAC6 complex, spin column chromatography (25) associated with LC–high-resolution mass spectrometry was used (Fig. 2*B*). When incubated with **1**, the predominant species that coeluted with zHDAC6-CD2 was a hydrated form of **1** (detected as **2**), which was present in a sevenfold molar excess over **3**. Both species were also detected in the fractions containing free compounds. Interestingly, **1** did not coelute with the enzyme. Furthermore, the addition of **2** as is to zHDAC6-CD2 failed to produce its stable chromatographically isolatable complex (Fig. 2*B*). Taken together, these data indicate that the complex with **1** itself and the hydrazide **3** observed in the crystal structure are unlikely to be the long-lived enzyme–inhibitor complex detected kinetically. Our data instead suggest that the high-affinity species is either the closed hydrated intermediate (Fig. 3, C1) or the *in situ* formed acyl hydrazide **2** (Fig. 3, C2).

In the first case, the tight complex would be formed by trapping a hydrated oxadiazole in the active site of HDAC6, which is generated as an intermediate upon nucleophilic attack by the Zn-bound water molecule. The second possibility is that the tight complex is formed with **2** but only when generated in the active site. In fact, formation of **2** starting from active site–bound **1** would lead to a complex in which the Zn-bound water molecule has reacted with the inhibitor. Upon addition to the free enzyme, **2** would instead encounter a second water molecule, which would need to be displaced. In this case, the hydrolysis reaction to yield **3** may be favored over the displacement of water molecule.

To gain more insight into the nature of the slow onset of inhibition observed with **1**, we performed experiments in D_2_O, reasoning that if the hydration of the DFMO ring is rate limiting, a solvent isotope effect should be observable. A significant deuterium solvent isotope effect on *k*_cat_ was detected when Fluor-De-Lys Green substrate was used (Table S3). However, we failed to observe an isotope effect on either the apparent association or the dissociation rate constants of **1** (Table 2), indicating that conformational changes, rather than chemical steps involving proton transfer reactions, are likely to be rate limiting.

To further characterize the mechanism, we introduced the Y745F and H574A mutations in the context of zHDAC6-CD2. Y745 is thought to orient the substrate carbonyl and to stabilize the tetrahedral intermediate in the deacetylation reaction of the acetyl-lysine substrate, and His574 is reported to act as a general base in the physiological hydrolytic reaction (12, 13). The onset of inhibition with **1** (Table 2) was significantly accelerated and too fast to be quantified upon manual mixing. Accordingly, the dissociation rate constant was about one order of magnitude greater in the Y745F mutant compared with the WT enzyme (Table 2). These results are indicative of a key role of Tyr745 in the observed rate-limiting conformational changes and in the stabilization of the tight complex.

To shed light on the roles of these key residues in DFMO hydration, we performed LC–MS experiments with both mutant forms (Fig. 2*A*). The observation that hydrolysis of **1** and **2** took place at a very low rate when incubated with zHDAC6-CD2 H574A further supports the conclusion that the hydrolytic reactions are enzyme catalyzed and involve H574 (Figs. 2*A* and S5). The fast hydrolysis rates of **1** measured with the Y745F mutant are likely the result of the lack of formation of the postulated tight complex and/or the exploitation of a different catalytic mechanism (Figs. 2*A* and S5). Furthermore, also the conversion of **2** into **3** was accelerated in this mutant (Fig. S5).

Finally, we found that the DFMO ring of **1** was also hydrolyzed by HDAC3 (a class I representative) and HDAC9 (a member of class IIa proteins), although at a slow rate and at very high inhibitor concentrations, leading to the formation of detectable levels of both **2** and **3** (Fig. S6). This finding indicates that the two-step hydrolytic reaction of this class of compounds is a common property associated with the overall architecture of HDAC active sites, which could be potentially exploited to develop novel subtype-selective inhibitors of other HDAC subtypes.

Figure 3 summarizes the reaction pathway, considering the modeling, crystal structure, and kinetic results.

## Conclusions

Our results indicate that DFMOs are mechanism-based inhibitors of HDAC6 that react with the enzyme as exquisitely selective substrate analogs, leading to *in situ* hydration and formation of a tight inhibitory reaction intermediate followed by the release of a ring-opened reaction product. The formation of a long-lived intermediate is at the origin of their potency. To the best of our knowledge, substrate analog inhibitors of hydrolases are unprecedented. We note however, that inhibition of steroid 5α-reductase by finasteride conforms to a similar mechanism and has led to the development of an approved drug (26), indicating that this mechanism can be exploited pharmacologically. DFMOs have excellent *in vitro* and *in vivo* drug-like properties and are promising candidates for the clinical development of HDAC6is for the treatment of several pathologies, such as peripheral neuropathies and for the modulation of immune responses. DFMOs are nontoxic, nonmutagenic, induce selective tubulin acetylation in cells, are metabolically stable, and reach *in vivo* exposures that are orders of magnitude higher than those obtained with hydroxamic acids (9). In addition, they are active in *in vivo* models of neuropathy at well-tolerated doses. Those data will be described elsewhere.

We observed that, albeit very inefficiently, HDAC3, class I HDAC, and HDAC9, class IIa enzyme, were also able to catalyze the ring opening of **1**, raising the question of whether appropriately modified oxadiazole-based inhibitors could also be generated to selectively target other HDAC isoforms. In our chemical series, the difluoromethyl-1,3,4-oxadiazole warhead gives an important contribution toward HDAC6 selectivity, but trifluoromethyl-1,2,4-oxadiazoles have been described as class IIa selective HDACis (27). Further modifications of the oxadiazole core may therefore be explored in the attempt to generate next-generation subtype-selective HDACis.

In conclusion, we described a novel mechanism of HDAC inhibition that may have an impact on the development of completely new classes of subtype-selective inhibitors as novel drugs for the treatment of pathologies in which HDACs have shown to play important roles.

## Experimental procedures

Full experimental procedures including synthesis of compounds, enzymatic measurements, protein production (Table S4 and Fig. S7), and X-ray crystallography are provided in the supporting information.

## Data availability

The data supporting the findings of this study are available within the article and supporting information.

## Supporting information

This article contains supporting information (13, 16, 21, 22, 28, 29, 30, 31, 32, 33, 34, 35, 36, 37, 38).

## Conflict of interest

The authors declare that they have no conflicts of interest with the contents of this article.
