# Supplementary material for: Difluoromethyl-1,3,4-oxadiazoles are slow-binding substrate analog inhibitors of histone deacetylase 6 with unprecedented isotype selectivity
Source: J Biol Chem. 2022 Dec 15;299(1):102800. doi: 10.1016/j.jbc.2022.102800 (PMC9860109; doi:10.1016/j.jbc.2022.102800)
Supplement: Supporting information [file mmc1.docx]

SUPPORTING INFORMATION

[**Selective HDAC6 inhibitors** 2](#_Toc114558253)

[**Comparison of compound 1 vs hydroxamate-based HDAC6 inhibitors** 3](#_Toc114558254)

[**Crystallographic parameters** 4](#_Toc114558255)

[**Kinetic characterization** 5](#_Toc114558256)

[**Docking analysis and QM/MM calculations** 7](#_Toc114558257)

[**LC-MS analysis of compounds 1 and 2 incubated with zHDAC6-CD2 Y745F and H574A** 9](#_Toc114558258)

[**LC-MS analysis of compound 1 after incubation with HDAC3 and HDAC9** 10](#_Toc114558259)

[**Experimental procedures** 11](#_Toc114558260)

[**CHEMISTRY** 11](#_Toc114558261)

[**ENZYMATIC MEASUREMENTS** 15](#_Toc114558262)

[**PROTEIN PRODUCTION AND X-RAY CRYSTALLOGRAPHY** 18](#_Toc114558263)

**Selective HDAC6 inhibitors**

**Figure S1.** Examples of DFMO-bearing selective HDAC6 inhibitors recently reported by Chong Kun Dang (1)(2) and Takeda (3). **1** is described in the present work (Table 1)

**Figure S2.** Structure of four selective hydroxamate-based HDAC6 inhibitors

**Comparison of compound 1 vs hydroxamate-based HDAC6 inhibitors**

**Table S1.** Comparison of the selectivity of compound **1** with that of the hydroxamate-based inhibitors ACY-1215 (Ricolinostat), ACY-241 (Citarinostat), KA2507 and ITF3756 on HDAC enzymes

| **HDACs** | | **IC_50_, µM​** | | | | |
| --- | --- | --- | --- | --- | --- | --- |
|  |  | **Compound 1​** | **ACY-1215** | **ACY-241** | **KA2507** | **ITF3756** |
| **Class I** | **HDAC1​** | > 100​ | 0.220 ± 0.008 | 0.348 ± ​0.009 | 7.145 ± 0.756 | 0.520 ± 0.012 |
|  | **HDAC2​** | > 100​ | 0.497 ± 0.018 | 0.928 ± 0.068 | 12.31 ± 0.86 | 1.058 ± 0.098 |
|  | **HDAC3​** | > 100 | 0.081 ± 0.002 | 0.126 ± 0.002 | 3.161 ± 0.375 | 0.231 ± 0.005 |
|  | **HDAC8​** | > 100 | 0.482 ± 0.018 | 0.525 ± 0.029 | 0.661 ± 0.069 | 0.269 ± 0.014 |
| **Class IIb** | **HDAC6​** | 0.0077 ± 0.0003 | 0.0055 ± 0.0002 | 0.0073 ± 0.0002 | 0.0135 ± 0.0007 | 0.0035 ± 0.0001 |
|  | **zHDAC6-CD2​** | 0.0078 ± 0.0004 | 0.0081 ± 0.0002 | 0.0119 ± 0.0003 | 0.0033 ± 0.0007 | 0.0010 ± 0.0001 |
|  | **zHDAC6-CD1​** | 82.2 ± 7.7​ | 0.480 ± 0.042 | 0.743 ± 0.053 | 0.411 ± 0.039 | 0.0681 ± 0.0015 |
|  | **HDAC10​** | > 100 | 0.212 ± 0.006 | 0.391 ± 0.015 | 5.783 ± 0.309 | 0.476 ± 0.018 |
| **Class IIa** | **HDAC4​** | > 100 | 7.687 ± 0.376 | 8.371 ± 0.405 | 3.869 ± 0.096 | 1.261 ± 0.115 |
|  | **HDAC5     ​** | > 100​ | 3.933 ± 0.122 | 5.966 ± 0.542 | 1.451 ± 0.051 | 0.513 ± 0.009 |
|  | **HDAC7​** | > 100​ | 5.103 ± 0.192 | 5.912 ± 0.479 | 1.824 ± 0.075 | 0.537 ± 0.027 |
|  | **HDAC9         ​** | > 100​ | 40.33 ± 2.89 | 44.65 ± 4.75 | 2.166 ± 0.231 | 0.604 ± 0.040 |
| **Class IV   ​** | **HDAC11      ​** | > 100​ | > 100​ | > 100​ | > 100​ | > 100 |

HDAC reaction velocities were measured as detailed in the experimental section below, at 25 °C, in the presence of a fixed concentration of fluorogenic substrates, varying concentrations of the inhibitors, and catalytic concentrations of HDAC, which were added to initiate the reaction. The initial velocities measured for HDAC forms, except for HDAC6 and **1**, were used for the calculation of the percental residual activity used to determine the IC_50_ by fitting the data to Eq. 1. For HDAC6 forms and **1**, steady-state velocities (v_s_) were instead used. The calculated slope factor *s* of Eq. 1 was approximately 1 in all cases. No greater than 50% inhibition was observed at the highest **1** concentration tested (100 µM) with class I, IIa, IV HDACs and HDAC10 indicating that the IC_50_ value is greater than 100 µM.

**Crystallographic parameters**

**Table S2.** Statistics of data collection and refinement. Values in parentheses are for the highest resolution shell

| **Complex** | **zHDAC6-CD2-ITF5924 (1)** |
| --- | --- |
| **Beamline** | XRD2 (Elettra) |
| **Wavelength (Å)** | 1.283 |
| **Space group** | *P*2_1_ |
| **Cell dimensions: a, b, c (Å), a, b, g (°)** | 52.37 118.92 60.26, 90.00 93.87 90.00 |
| **Resolution (Å)** | 47.84-1.60 (1.63-1.60) |
| ***R*_merge_** | 0.133 (2.494) |
| **Mean I/sI** | 8.8 (0.8) |
| **Completeness (%)** | 95.6 (91.9) |
| **Redundancy** | 7.5 (7.4) |
| **Half-set correlation CC_1/2_ (%)** | 99.8 (37.0) |
| **Wilson *B* factor (Å^2^)** | 19.1 |
| **Final Refinement** | |
| **Resolution range** | 47.88-1.60 (1.64-1.60) |
| **No. of reflections** | 87601 (6589) |
| **Completeness (%)** |  |
| **No. of atoms** | 6377 |
| ***R*_work_/*R*_free_ (*R*_free_ = 5% of reflections)** | 0.16/0.19 (0.32/0.33) |
| **Mean *B*-value, overall (Å^2^)** | 23.7 |
| **RMSD from ideal values – bond length (Å)/bond angle (°)** | 0.01/1.61 |
| **Correlation coefficient between *F_o_* and *F_c_* all/free** | 0.98/0.97 |
| **Ramachandran plot (Coot) – favored/allowed/disallowed (%)** | 94.1/4.6/1.3 |

*R*_merge_ = ΣhklΣi|Ii,hkl − ⟨I⟩hkl|/ΣhklΣiIi,hkl, where ⟨I⟩hkl is the average intensity calculated for reflection hkl from replicate measurements. *R*_work_ = Σ||Fo| − |Fc||/Σ|Fo| for reflections contained in the working set. |Fo| and |Fc| are the observed and calculated structure factor amplitudes, respectively. *R*_free_ is calculated using the same expression for reflections contained in the test set held aside during refinement.

**Kinetic characterization**

**Table S3.** Solvent Kinetic Isotope Effects on the deacetylation reaction catalyzed by human HDAC6 and kinetic parameters exhibited by zHDAC6-CD2

| **Assay buffer** | **Fluor De Lys green** | | |
| --- | --- | --- | --- |
|  | **K_m_, µM** | **k_cat_, min^-1^** | **k_cat_/K_m_, µM^-1^ min^-1^** |
| **1** | 4.1 ± 0.1 | 13.1 ± 0.1 | 3.3 ± 0.1 |
| **2** | 4.7 ± 0.2 | 7.3 ± 0.1 | 1.5 ± 0.1 |
| **1**^a^ | 3.1 ± 0.2 | 15.7 ± 0.2 | 5.1 ± 0.3 |
| **2**^a^ | 2.5 ± 0.1 | 8.2 ± 0.1 | 3.2 ± 0.1 |

The initial velocities of reactions were measured by following the time-course of product formation in reactions containing varying concentrations of Fluor-de-Lys Green (0.78-50 µM) and human HDAC6 (0.125 nM) at 25 °C in buffer prepared in H_2_O (1) or D_2_O (2). Fluorescence changes were converted to changes in concentration of the deacetylated product using the deacetylated Fluor-de-Lys Green standard (Enzo Life Sciences). The steady-state kinetic parameters K_m_ for Fluor-de-Lys Green and k_cat_ were calculated by fitting the data to the Michaelis-Menten equation. ^a^Analogous experiments were performed with zHDAC6-CD2 wt.


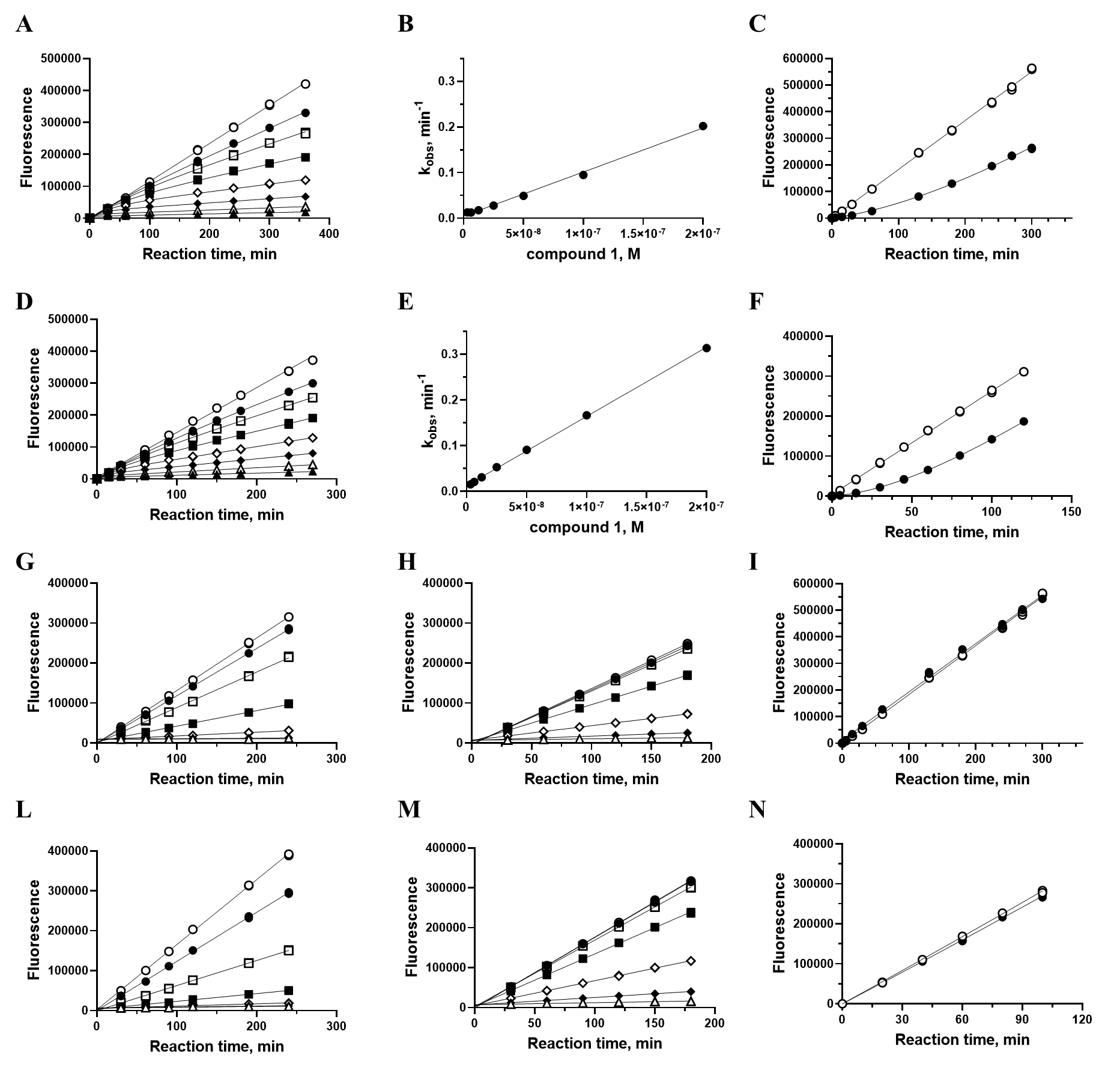


**Figure S3.** Effects of compounds **1**, **2** and **3** on human HDAC6 and zHDAC6-CD2

The progress curves for human HDAC6 (**A**) and zHDAC6-CD2 (**D**) in the presence of various concentrations of **1** were carried out in assay buffer and 3 µM Fluor-de-Lys Green. 0 (○), 3.125 (●), 6.25 (□), 12.5 (■), 25 (◇), 50 (◆), 100 (△) and 200 (▲) nM **1**. The curves were fitted with Eq 2. Replot of the calculated k_obs_ values for inhibition of HDAC6 (**B**) and zHDAC6-CD2 (**E**) as function of **1** concentration. HDAC6 (**C**) or zHDAC6-CD2 (**F**) were incubated in the absence (○) or in the presence of 100 nM **1** (●) for 120 min. The solutions were then rapidly diluted 100-fold by addition of 50 µM Fluor-de-Lys Green substrate in assay buffer. **(G)** 0 (○), 32 (●), 160 (□), 800 (■), 4000 (◇), 20000 (◆) and 100000 (△) nM **3** and HDAC6. **(H)** 0 (○), 32 (●), 160 (□), 800 (■), 4000 (◇), 20000 (◆) and 100000 (△) nM **2** and HDAC6. **(I)** Jump dilution assay with HDAC6. Control (○), **3** (30 nM, final) (●). **(L)** 0 (○), 32 (●), 160 (□), 800 (■), 4000 (◇), 20000 (◆) and 100000 (△) nM **3** and zHDAC6-CD2. **(M)** 0 (○), 32 (●), 160 (□), 800 (■), 4000 (◇), 20000 (◆) and 100000 (△) nM **2** and zHDAC6-CD2. **(N)** Jump dilution assay with zHDAC6-CD2, control (○), **2** (2.5 nM final) (●). All the IC_50_ values and calculated kinetic constants are summarized in Tables 1 and 2.

**Docking analysis and QM/MM calculations**

All docking calculations were performed by using the Glide module(4) of the Schrödinger suite 2021-3, by using the zHDAC6-CD2 structure (PDB code 7O2R).

Ligands were prepared using LigPrep (5) (Schrödinger) by modifying the torsions of the ligands and assigning them appropriate protonation states [Glide (4)-Schrödinger, XP extra precision]: a single stereochemical structure was generated per ligand with possible states at target pH 7.0 ± 2.0 using Ionizer and Epik (6) by adding metal binding states, tautomerized, desalted and optimized by producing low energy 3D conformation for the ligand under the Optimized Potentials for Liquid Simulations (OPLS-2005) force field while retaining the specified chiralities of the input ligand.

The QSite module (7) was adopted for QM/MM refinement of docking best poses and hydrolyzed intermediates derived from the catalytic mechanism of HDAC6 recently proposed (8), adopting DFT/B3LYP method with the 6-31g*/lacvp basis set. The QM/MM boundary was treated by a hydrogen cut, with the ligand-enzyme complex region restricted to the zinc cation, free ligands (water and DFMO-based inhibitor, or hydrated DFMO) and sidechains residues in a sphere with a radius equal to 5 Å (Phe583, Phe643, His573, His574, Asp612, His614, Asp705, Tyr745, Cys584 zHDAC6-CD2; Asp175, Asp264, His178, His140, His141, Tyr303, Phe205, Phe150, Cys151, h-HDAC1, PDB code 5ICN; Phe202, Trp261, His192, His193, Asp230, His232, Asp323, Tyr363, Cys203, zHDAC6-CD1, PDB code 5G0I) were treated at DFT/B3LYP level of theory, with the 6-31g*/lacvp basis set.

It is worth to note that several ligands co-precipitated with zHDAC6-CD1 are currently available:

Trichostatin A (PDB codes 6UO2, 5G0G, 6UO4, 5EEF), AR-42 (PDB codes 6UO3, 6UO5, 6UO7), Resminostat (PDB code: 6UOB), Givinostat (PDB code 6UOC). However, all compounds are pan-inhibitors, whereas only one crystal structure (PDB code 5G0I) between CD1 and a selective HDAC6 inhibitor (Nexturastat A) is accessible.

**Figure S4.** Water distances in zHDAC6-CD2 (A), h-HDAC1 (B) and zHDAC6-CD1 (C) optimized geometries (QM/MM method). In HDAC1, oxygen atom is closer to oxadiazole centroid than aromatic electrophilic carbon atom, the opposite condition revealed by HDAC6 calculation, suggesting that solvent unshared electron pair could be trapped in n→π* interaction. Geometry detected in CD1 is intermediate but zinc cation i) interacts with delocalized π cloud of five membered ring, instead of the lone pair localized on the closest Nitrogen atom; ii) is far away from water molecule, suggesting a weaker bond. Superposition of final QM/MM conformers of inhibitor in CD1 and CD2 indicates that ZBG penetrates 1.45 Å deeper in the catalytic core (structure with carbon atoms in green color, panel D), whereas water molecule and zinc exhibits a less pronounced displacement. A similar behavior (E) is detectable in complex between Nexturastat A and zHDAC6 (PDB code 5G0I), although the cation is bound to a different ZBG and the effect is less significant (0.63 Å), binding conformer in CD1 (carbon atoms in green color) is buried more deeply. Superposition (F) of complexes between Nexturastat A in CD1 (carbon atoms in green color) and CD2 (carbon atoms magenta-colored) suggests a different effect played by K330 and L712 in CD1 and CD2, respectively: L712 is the main component of a hydrophobic pocket able to accommodate the aromatic moiety of inhibitor cap-term, whereas flexibility of K330 side chain hinders docking of phenyl substructure, pushing cap-term 1.97 Å away.


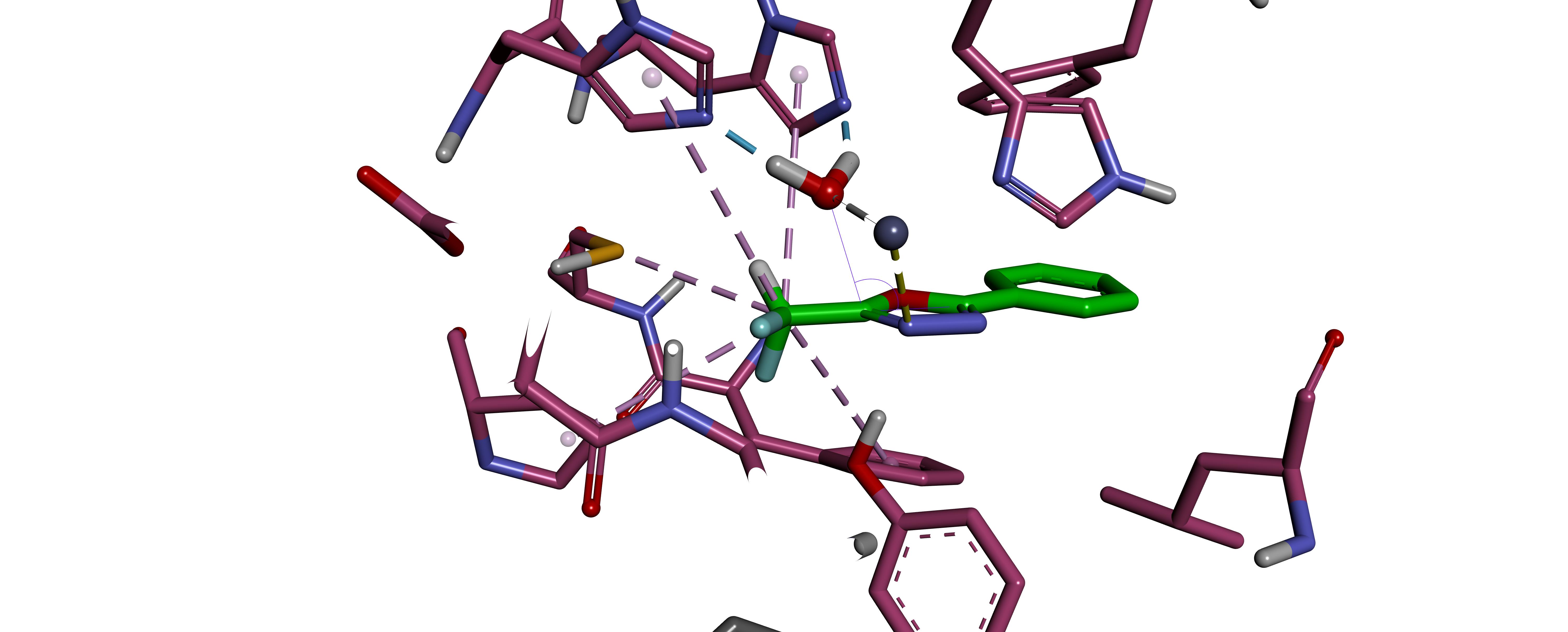


A


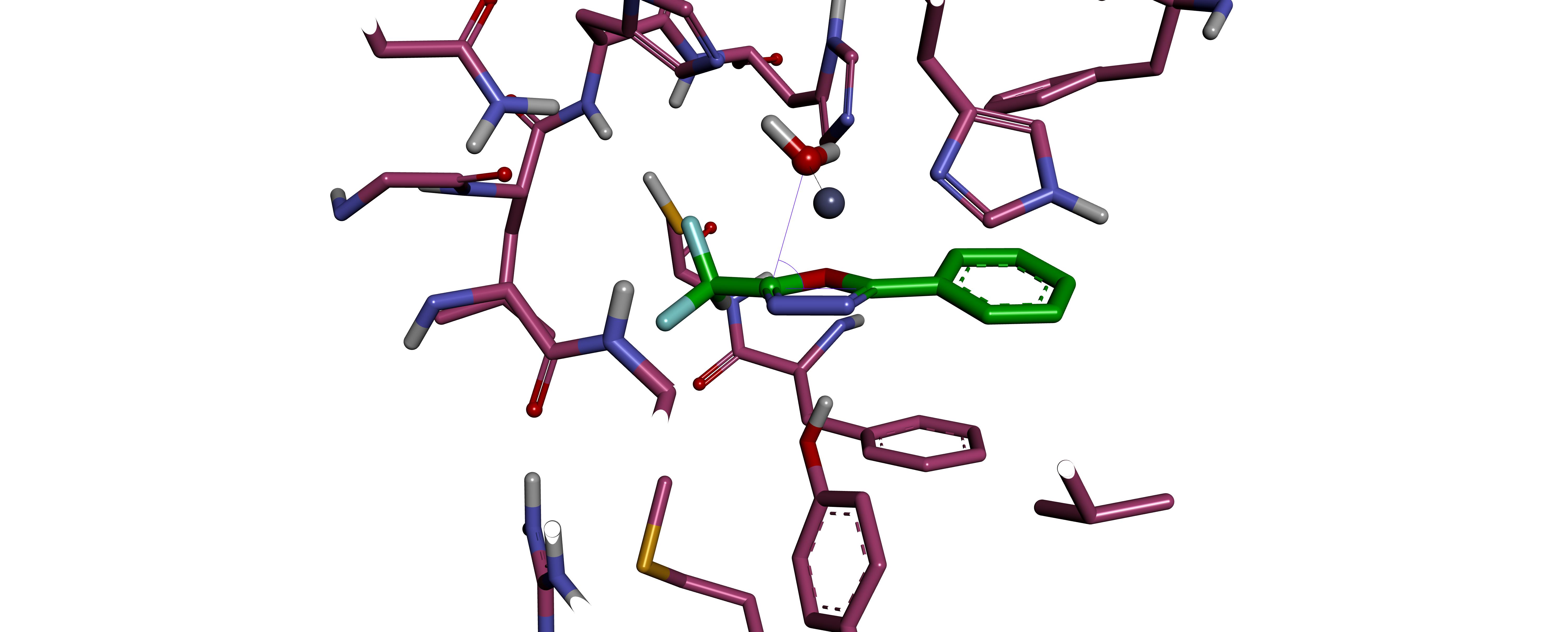


B

**71.5^o^**

**3.07 Å**

**2.12 Å**

**114^o^**

**2.79 Å**

**2.08 Å**


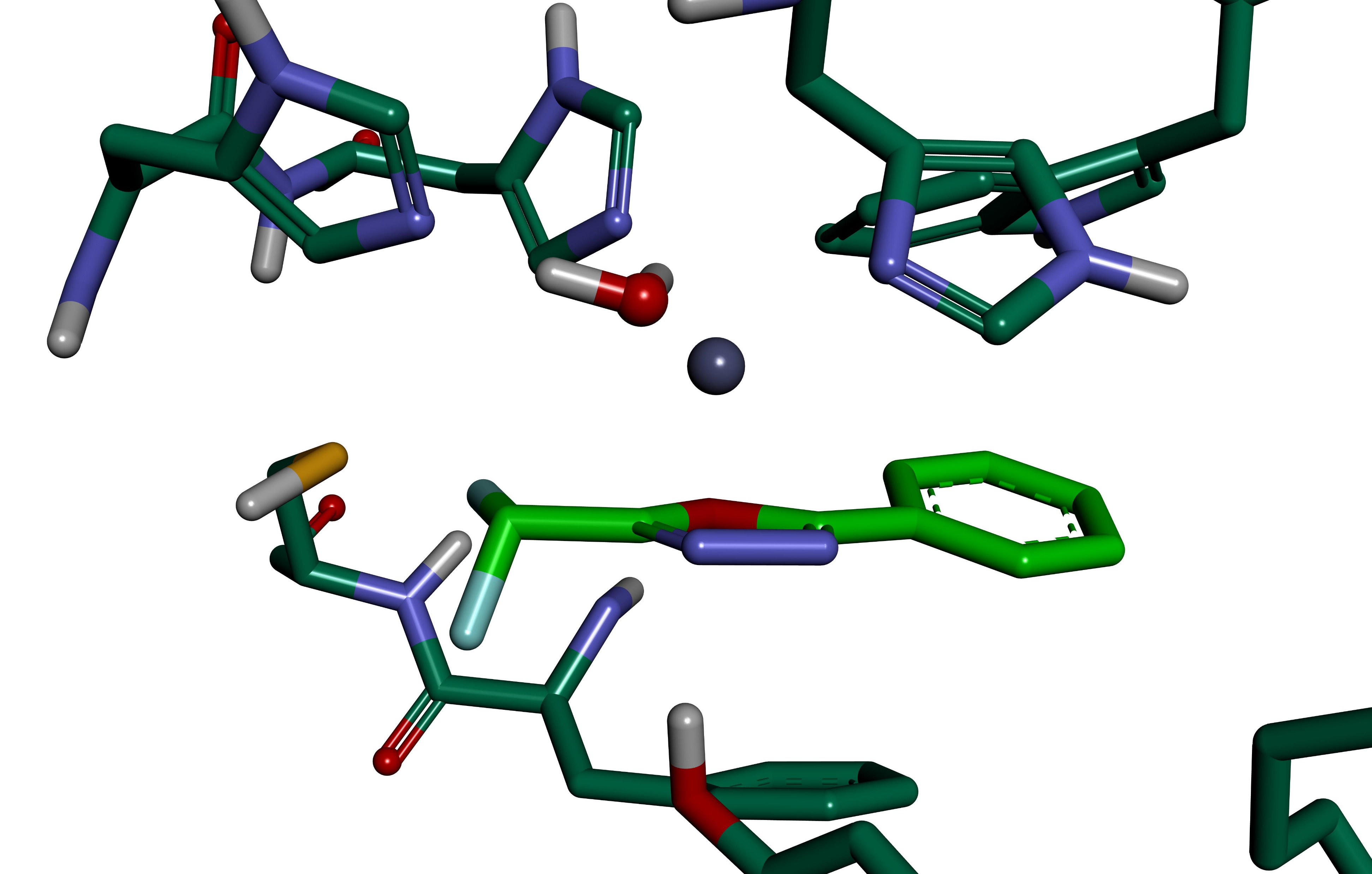


**94.9^o^**

**2.70 Å**

**2.25 Å**

C

D


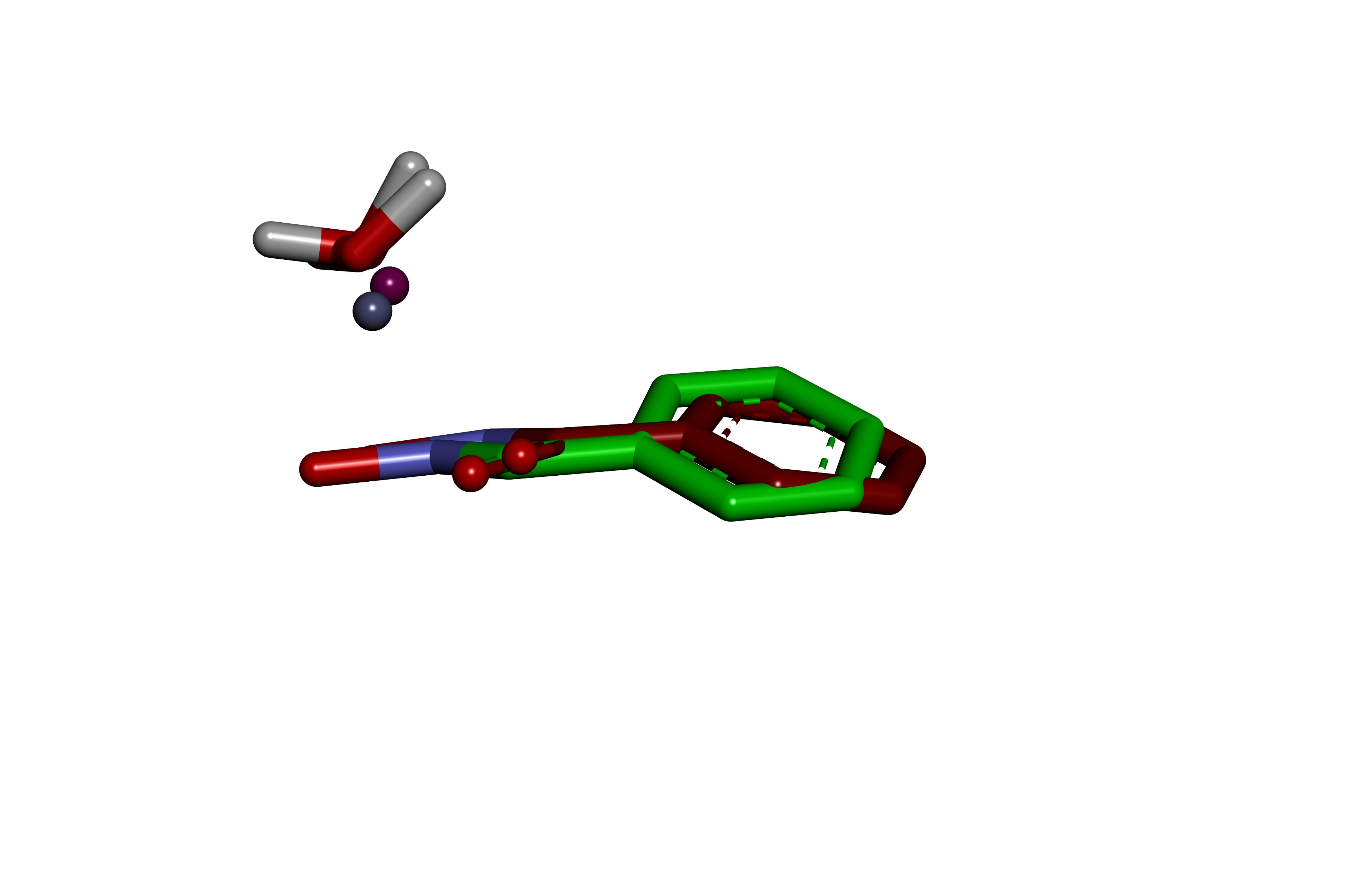


**0,63 Å**

E


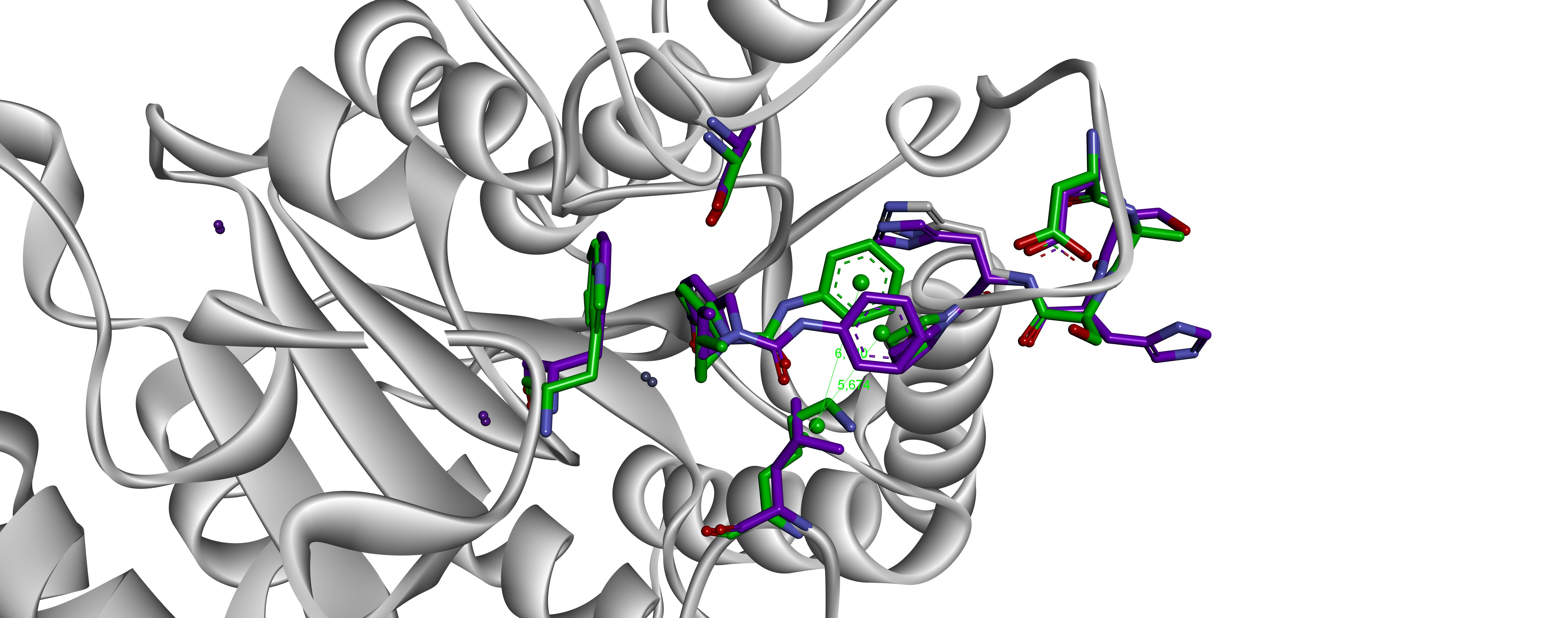


F


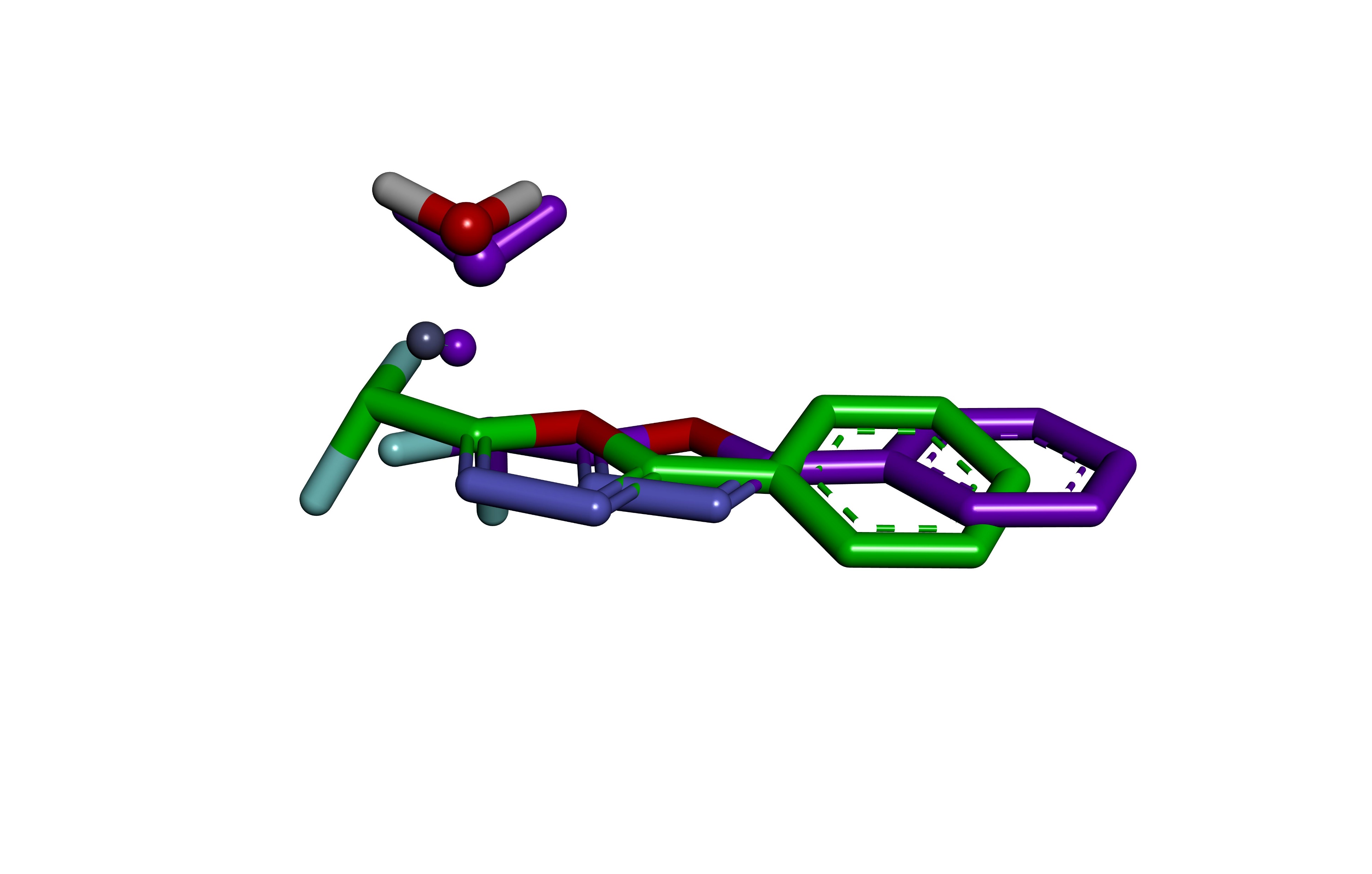


**1.45 Å**

**LC-MS analysis of compounds 1 and 2 incubated with zHDAC6-CD2 Y745F and H574A**


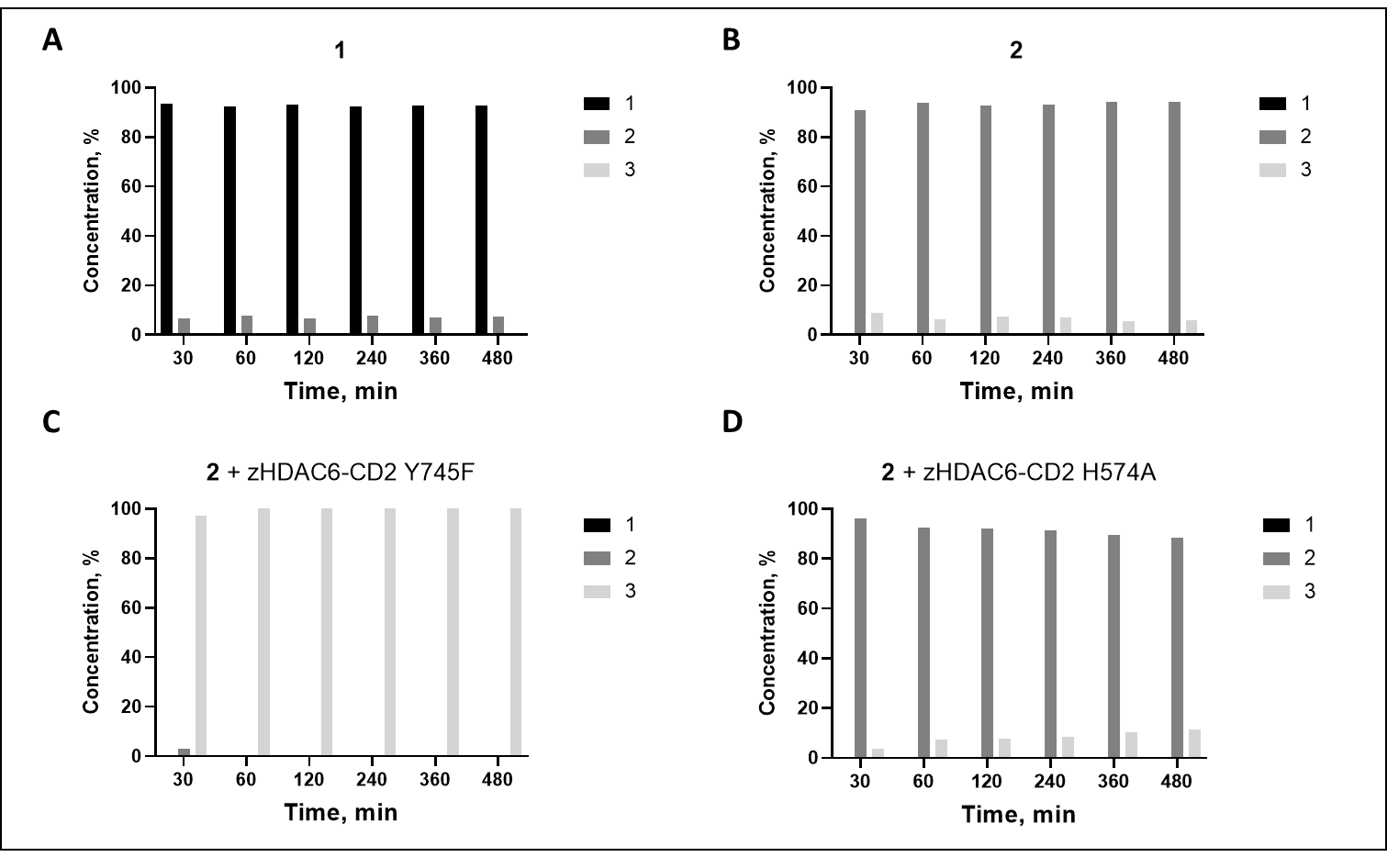
**Figure S5.** The hydrolysis of compounds **1** and **2** with or without zHDAC6-CD2 mutants

5 µM **1** (**A**) or **2** (**B**) were incubated in assay buffer devoid of enzymes at 25 °C for up to 8 hours. At different times an aliquot was withdrawn and analyzed by LC-HRMS for the quantification. Experiments similar to those described in **Figure 2A** (main text) were carried out by incubating **2** with 1 µM zHDAC6-CD2 Y745F (**C**) or zHDAC6-CD2 H574A (**D**).

**LC-MS analysis of compound 1 after incubation with HDAC3 and HDAC9**


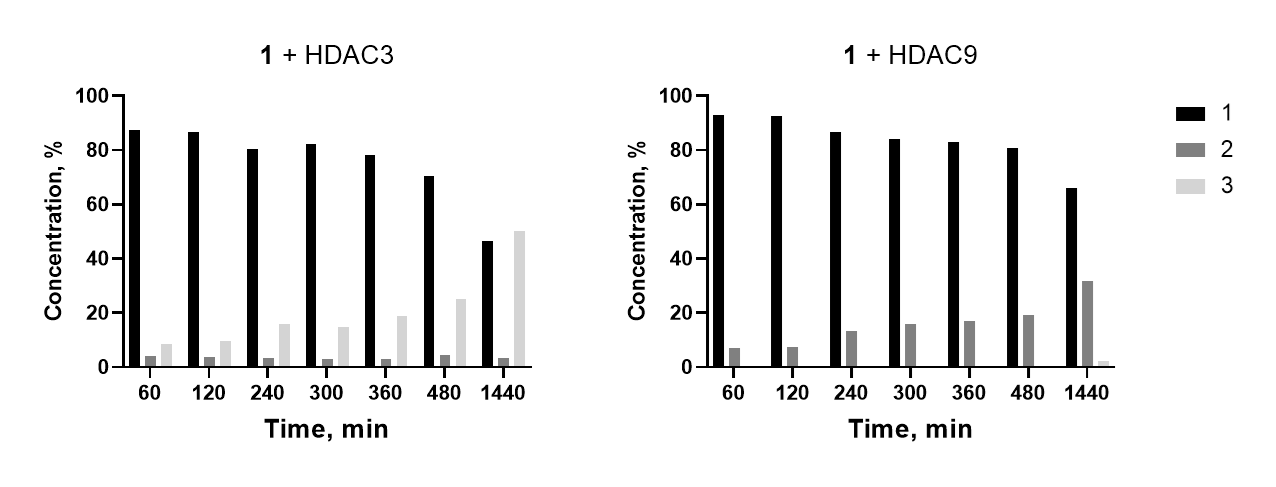


**Figure S6.** The hydrolysis of compound **1** by HDAC3 and HDAC9

**1** (100 µM) was incubated in the presence of HDAC3 (1 µM) or HDAC9 (1 µM) at 25 °C under the conditions of the experiments shown in **Fig 2A** (main text) and **S6**. At different times, aliquots were withdrawn for quantification of **1** and its derivatives **2** and **3** by LC-HRMS.

**Experimental procedures**

**CHEMISTRY**

**Synthesis of N-{4-[1-({4-[5-(difluoromethyl)-1,3,4-oxadiazol-2-yl] phenyl} methyl)-1H-1,2,3-triazol-4-yl] phenyl}-4,5-dihydro-1H-imidazol-2-amine (compound 1).**

Step A

Methyl 4-methylbenzoate (70 g, 466 mmol, 1 equiv.) was dissolved in 350 mL MeOH, then hydrazine monohydrate was added (2.5 equiv.) under stirring. Mixture was refluxed overnight. Full conversion of methyl ester to hydrazide was observed by LCMS (and TLC). The reaction mixture was concentrated and dried under vacuum. The white solid obtained (70 g, 99% yield) was used for the subsequent step without further purification.

Step B

 

Hydrazide obtained in step A (50 g, 333 mmol, 1 equiv.) was dissolved in dry DMF (300 mL) under argon. Difluoroacetic anhydride (3 equiv.) was slowly added through a dropping funnel, keeping temperature below 10 °C (ice/NaCl bath). After addition was complete, the temperature was let to reach room temperature (r.t.). The flask was sealed, and the reaction mixture was stirred at r.t. overnight. Conversion was observed by LCMS.

The reaction mixture was carefully poured into sat. aq. NaHCO_3_ to quench DFAA excess. The product precipitated as a solid and could be collected by filtration. After removing residual solvents, the crude product was dissolved in MTBE, washed 3 times with 300ml of NaHCO_3_ and brine. Organic phase was dried over Na_2_SO_4_, filtered, and evaporated to give a crude product (43 g, 205 mmol, 61% yield), which was used in the next step without further purification.

Step C

 

2-(difluoromethyl)-5-(4-methylphenyl)-1,3,4-oxadiazole (43 g, 205 mmol, 1 equiv.) was dissolved in 800 ml degassed carbon tetrachloride.  *N*-Bromosuccinimide (NBS, 1.05 equiv.) and azobisisobutyronitrile (AIBN, 0.01 equiv.) were added to the reaction mixture, which was stirred at 75 °C over 4 h.

The mixture was diluted with DCM, washed with water (3x200 mL), NaHCO_3_ (200 mL) and brine (2x200 mL). Organic phase was dried over Na_2_SO_4_, filtered, and evaporated to give a crude product (80 g). Crude residue was reprecipitated from DCM/hexane, affording pure product (36 g, 125 mmol, 61% yield).

Step D

A solution of 2-[4-(bromomethyl)-phenyl]-5-(difluoromethyl)-1,3,4-oxadiazole (1 g, 3.46 mmol, 1 equiv.) and sodium azide (1 equiv.) in 10 mL DMSO was stirred at r.t. for 1 h. Full conversion to intermediate 2-(4-(azidomethyl)-phenyl)-5-(difluoromethyl)-1,3,4-oxadiazole was observed by HPLC.

4-Ethynylaniline (405 mg, 1 equiv.) was then added to the reaction mixture, followed by copper(II) sulfate pentahydrate (0.2 equiv., 0.5 M aqueous solution) and sodium l-ascorbate (0.4 equiv., 1 M aqueous solution). The reaction mixture was agitated at 40 °C overnight. Full conversion of the starting material was detected by LC-MS. Water was added to the reaction mixture, and a precipitate formed. The precipitate was filtered and washed with water. Purification by flash chromatography (silica gel, Hex:EtOAc 0-100%) gave the desired product in good purity (973 mg, 2.61 mmol, 75% yield).

Step E

HgCl_2_ (1.1 equiv.) was added to a solution of 4-(1-(4-(5-(difluoromethyl)-1,3,4-oxadiazol-2-yl)benzyl)-1H-1,2,3-triazol-4-yl)aniline (84 mg, 0.228 mmol, 1 equiv.), N,N'-di(*tert*butoxycarbonyl)imidazolidine-2-thione (1 equiv.) and triethylamine (1.3 equiv.) in 1 mL DCM at 0 °C. The resulting mixture was stirred at 0 °C for 1 h and at r.t. for 2 days. The reaction mixture was diluted with water and DCM, filtered and extracted with DCM. The organic layer was washed with brine, dried over MgSO_4_, filtered, and concentrated under reduced pressure to afford a yellow oil, which was used directly in the next step (145 mg, 0.228 mmol, 100% yield).

Step F

Di-*tert*-butyl 2-((4-(1-(4-(5-(difluoromethyl)-1,3,4-oxadiazol-2-yl)benzyl)-1H-1,2,3-triazol-4-yl)phenyl)imino)imidazolidine-1,3-dicarboxylate (145 mg, 0.228 mmol, 1 equiv.) was dissolved in 2 mL DCM and TFA (20 equiv.) was added. The reaction mixture was stirred at r.t. overnight. The mixture was diluted with DCM and washed with sat. aq. NaHCO_3_ and brine. During washing with brine, precipitation occurred. The solid was filtered, washed with water and dried under vacuum to obtain the desired product (55 mg, 0.121 mmol, 53% yield). 1 H-NMR (400 MHz, DMSO) δ 8.75 (s, 1H), 8.20 (d, 2H), 7.90 (d, 2H), 7.60 (s, 2H), 7.55 (t, 1H), 7.30 (d, 2H), 5.78 (s, 2H), 3.65 (s, 4H). [M + H]^+^ = 437.12.

**Synthesis of N'-(2,2-difluoroacetyl)-4-((4-(4-((4,5-dihydro-1H-imidazol-2-yl)amino)phenyl)-1H-1,2,3-triazol-1-yl)methyl)benzohydrazide (compound 2).**

N-[4-[1-[[4-[5-(difluoromethyl)-1,3,4-oxadiazol-2-yl]phenyl]methyl]triazol-4-yl]phenyl]-4,5-dihydro-1H-imidazol-2-amine (50 mg, 0.115 mmol, 1 equiv.) was suspended in 1 mL of a 1:1 water/ACN mixture and TFA (40 equiv.) was added. The reaction mixture was stirred at r.t. overnight. Full conversion of the starting material was observed by LCMS.

The reaction mixture was purified by prep HPLC (ACN + 0.1% FA /H_2_O + 0.1% FA) affording 20 mg of the desired product (0.043 mmol, 37% yield). 1 H-NMR (400 MHz, DMSO) δ 10.25 (s, 4H), 8.60 (s, 1H), 8.30 (s, 1H, HCOOH), 7.80 (dd, 4H), 7.45 (d, 2H), 7.25 (d, 2H), 6.30 (t, 1H), 5,75 (s, 2H), 3.55 (s, 4H). [M + H]+ = 455.23.

**Synthesis of 4-((4-(4-((4,5-dihydro-1H-imidazol-2-yl)amino)phenyl)-1H-1,2,3-triazol-1-yl)methyl)benzohydrazide (compound 3).**

Step A

A solution of 4-(bromomethyl)benzoic acid (1 g, 4.65 mmol, 1 equiv.) and sodium azide (1 equiv.) in 20 mL DMF was stirred at r.t. for 1 h. HATU (1.1 equiv.), *N,N*-diisopropylethylamine (2 equiv.) and tert-butyl hydrazinecarboxylate (1 equiv.) were added; the reaction mixture was stirred at r.t. overnight. Conversion of the starting material was monitored by LCMS. Copper(II) sulfate pentahydrate (0.15 equiv., 0.5 M aqueous solution) and sodium l-ascorbate (0.3 equiv., 1 M aqueous solution) were added. The reaction mixture was stirred at r.t. overnight; full conversion to the desired product was observed by LCMS.

Upon dilution with water the product precipitated as a solid (1.76 g, 4.32 mmol, 92% yield) which was used directly in the next step.

Step B

HgCl_2_ (1.1 equiv.) was added to a solution of tert-butyl 2-(4-((4-(4-aminophenyl)-1H-1,2,3-triazol-1-yl)methyl)benzoyl)hydrazine-1-carboxylate (786 mg, 1.92 mmol, 1 equiv.), di*tert*-butyl 2-sulfanylideneimidazolidine-1,3-dicarboxylate (1.1 equiv.) and triethylamine (3 equiv.) in 10 mL DCM. The reaction mixture was stirred at r.t. over 48 h. Full conversion of the starting material was detected by LCMS.

The reaction mixture was diluted with DCM and filtered on a Celite pad. The filtrate was washed with brine (3x), dried over Na_2_SO_4_, filtered, and concentrated under reduced pressure to afford a yellow/orange solid (1.30 g, 1.92 mmol, 99% yield) which was used in the next step without any further purification.

Step C

Di*tert*-butyl2-[4-[1-[[4-[[(2-methylpropan-2-yl)oxycarbonylamino]carbamoyl]phenyl]methyl]triazol-4-yl]phenyl]iminoimidazolidine-1,3-dicarboxylate (650 mg, 0.96 mmol, 1 equiv.) was dissolved in 4 mL DCM and TFA (30 equiv.) was added. The reaction mixture was stirred at r.t. overnight. The full conversion of the starting material was monitored by LCMS.

The reaction mixture was neutralized with NaHCO_3_; the organic solvent was removed under reduced pressure. The crude residue was purified by RP Prep-HPLC (H_2_O/ACN: 95/5 to 80/20). The desired product was isolated as a white solid (140 mg, 0.37 mmol, 38% yield). 1H NMR (400 MHz, DMSO) δ 9.75 (s, 1H), 8.75 (s, 1H), 8.30 (s, 2H), 7.80 (dd, 4H), 7.30 (dd, 4H), 5.70 (s, 2H), 4.50 (s, 2H), 3.70 (s, 4H). [M + H]+ = 377.23.

**ENZYMATIC MEASUREMENTS**

**HDAC activity assays**

Recombinant human HDACs (HDACs 1-11) and zebrafish HDAC6 forms used in this study were purchased from vendors as detailed in the **Table S4**.

HDAC activity was determined by monitoring the deacetylation of the fluorogenic substrates: Fluor-de-Lys Green (for HDAC1, 2, 3, 6, 8 and 10, Enzo Life Sciences), Nε-Trifluoroacetyl-L-lysine-AMC (for HDAC4, 5, 7, 9 and zHDAC6-CD2 Y745F, in-house synthesis) and Ac-ETDK(myristoyl)-AMC (for HDAC11, GenScript). Unless otherwise stated reactions were carried out at 25 °C in 25 mM Tris/HCl, pH 8.0, 130 mM NaCl, 0.05% Tween-20, 10% Glycerol, 1 mg/mL BSA, 0.5 mM tris(2-carboxyethyl)phosphine (TCEP) and 1% DMSO (assay buffer). Human HDAC forms were used at catalytic concentrations of 710 pM, 650 pM, 115 pM, 18 pM, 300 pM, 62.5-125 pM, 80 pM, 5 nM, 120 pM, 10 nM, and 5 nM. The concentrations of zebrafish forms were 1 nM (zHDAC6-CD1), 0.12-0.5 nM (zHDAC6-CD2 wt) and 0.5 nM (zHDAC6-CD2 Y745F). The reactions were started by addition of enzyme to the assay mixtures equilibrated at 25°C in test tubes in a final volume of 500-1000 µL. At different times, aliquots (110 µL) were withdrawn and transferred to test tubes containing an equal volume of “Stop/Developer solution” consisting of the commercial Fluor-de-Lys Green Developer (Enzo Life Sciences) and 2-20 µM of HDAC class-selective inhibitor. After incubation at r.t. for at least 25 min to allow for the release of the fluorophore from the deacetylated reaction product, aliquots (100 μl) were transferred in technical duplicate to white polystyrene 96-well Half-Area plates (PerkinElmer) for fluorescence intensity measurements using a multilabel plate reader (Victor, PerkinElmer). For inhibition studies, the substrates were included at a concentration close to the K_m_ value, except for class IIa HDACs, zHDAC6-CD1 and HDAC8, where the used concentrations of substrate were about 10-fold lower than the value of K_m_. The inhibitor concentrations were selected to vary them between approximately 0.1 and 10-fold the IC_50_ value. For HDAC/inhibitor couples yielding a linear increase of product formation during the reaction, the initial velocity (v_o_) was calculated as (∆F/min) from fitting the fluorescence values to a straight line. In the case of HDAC6 and **1**, the progress curves were curvilinear indicating a slow onset of inhibition. Thus, the data were fitted to Eq 2 to obtain the initial velocity (v_i_) and the steady-state velocity at long reaction times, where the slow equilibrium is established between free enzyme and inhibited forms (v_s_), and the apparent rate of conversion of the inhibited complex in equilibrium with free enzyme and inhibitor (k_obs_). In Eq 2, P is the product concentration, expressed as fluorescence intensity and t is the reaction time (in minutes). The initial velocity values (v_i_) appeared to be similar to that of the enzyme in the absence of inhibitor, suggesting that no rapid equilibrium is established between the enzyme and the inhibitor or that the inhibition constant for such process is very high. Therefore, the initial velocity (v_i_) was often set to the value obtained in control reactions in the absence of inhibitor. For IC_50_ determination, the initial velocity value (v_o_) for fast-on inhibitors or the steady-state velocity values (v_s_) for **1** expressed as (∆F/min) were used to calculate the residual activity, as percentage with respect to the value obtained in the absence of inhibitor. Such percental residual activity (y) as a function of inhibitor concentration (x) was fitted with Eq 1 to obtain estimates of IC_50_ and of the slope factor (s), corresponding to the Hill coefficient, and of their associated error. In all cases the slope factor s was approximately 1.

Eq. 1 y = 100/[1+(x/IC_50_)^s^]

Eq. 2 P = v_s_*t + (v_i_ - v_s_)[1 - exp(-k_obs_*t)]/k_obs_

For the slow onset of inhibition of HDAC6 forms in the presence **1**, the dependence of the k_obs_ values on inhibitor concentration was used to determine the mechanism of inhibition. The observed linear dependence indicates a one-step process of the type E + I ↔ EI, which is well described by Eq 3, where the apparent rate of formation of the inhibited complex EI (k_on_^app^) and of release of the inhibitor from the complex (k_off_^app^) determine k_obs_. As an alternative, a linear plot of k_obs_ as a function of the inhibitor may reflect a two-step process of the type E+I ↔ EI ↔ EI*. First, an EI complex is formed in rapid equilibrium with free enzyme and inhibitor. Then, such initial EI complex is slowly converted into a tighter EI* complex, but the dissociation constant of the first EI complex K_i_ is very large. Thus, v_i_ approximates v_o_, and IC_50_ reflects the dissociation constant of the EI* complex (Ki*). In the latter case, the slope of the line is the apparent association rate constant describing the formation of the EI* complex, whereas the intercept reflects the rate of dissociation of the EI* complex.

Eq. 3 k_obs_ = k_on_^app^ [I] + k_off_^app^

So-called jump-dilution assays (9) were used to establish the reversibility of the enzyme-inhibitor complex that is slowly formed between HDAC6 forms and **1**. Enzymes were pre-incubated for 60-120 minutes with **1**, **2**, or **3** at a concentration at least 10 times greater than IC_50_ value. The enzyme-inhibitor solution was then rapidly diluted 100- to 2000-fold into assay mixtures containing saturating concentrations of substrates (50 µM Fluor-de-Lys Green for hHDAC6 and zHDAC6-CD2 wt or 25 µM TFAL for zHDAC6-CD2 Y745F) to start the reaction. Final concentrations of enzymes were 0.15 nM hHDAC6, 0.15-0.5 nM zHDAC6-CD2 and 0.5 nM zHDAC6-CD2 Y745F. The recovery of enzyme activity was followed as a function of reaction time as already described. Progress curves of product formation were fitted with Eq 2, where the initial velocity (v_i_) is now the initial velocity of the fully inhibited enzyme; steady-state velocity (v_s_) is the velocity measured when a steady-state is established and approached that measured in the absence of inhibitor; k_obs_ is the apparent first-order rate constant of dissociation of the enzyme-inhibitor complex to yield catalytically active enzyme (k_off_) under conditions that minimize inhibitor rebinding (slow onset of inhibition and substrate concentration well above K_m_ value).

Solvent kinetic isotope effects (SKIE) were determined in 25 mM Tris/HCl, pH 8.0, 130 mM NaCl, 0.05% Tween-20, 10% glycerol, 1 mg/mL BSA, 0.5 mM TCEP and 1% DMSO and 125 pM HDAC6 with all buffers and stock solutions prepared in H_2_O or D_2_O. No correction of pD was applied according to a recent reported work (10), and kinetic data obtained in this buffer (pD = 8) were compared to the ones carried out in standard assay buffer (pH = 8). The initial velocities of the reactions were measured by monitoring the time-course of product formation in the presence of 0.78-50 µM Fluor-de-Lys Green. The steady-state kinetic parameters (K_m_ and k_cat_) were calculated by fitting the data with the Michaelis-Menten equation that also provides estimates of the error associated with the parameters. The inhibition studies were carried out in protiated or deuterated assay buffers containing 3 µM Fluor-De-Lys Green, 0.125 nM HDAC6 and 3.12-200 nM **1**, a control reaction devoid of inhibitor was also set-up.

**Table S4.** Recombinant HDAC proteins

| **Recombinant proteins** | **Tag** | **Expression system** | **Source​** | | **Purity** | **Identifier​** |  |
| --- | --- | --- | --- | --- | --- | --- | --- |
| Human HDAC1, full length | C-terminal His-tag and FLAG tag | Baculovirus infected Sf9 cells | BPS Bioscience | | ≥ 79 % | Cat # 50051, Lot # 181108-1 |  |
|  |  |  |  |  |  |  |  |
| Human HDAC2, full length | C-terminal His-tag | Baculovirus infected Sf9 cells | BPS Bioscience | | ≥ 88 % | Cat # 50002, Lot # 160701 |  |
|  |  |  |  |  |  |  |  |
| Human HDAC3/NcoR2, full length | C-terminal His-tag | Baculovirus infected Sf9 cells | BPS Bioscience | | ≥ 80 % | Cat # 50003, Lot # 130819 |  |
|  |  |  |  |  |  |  |  |
| Human HDAC4, a.a. 627-1084 (end) | N-terminal GST-tag,  C-terminal His-tag | Baculovirus infected Sf9 cells | BPS Bioscience | | ≥ 89 % | Cat # 50004, Lot # 130828-G |  |
|  |  |  |  |  |  |  |  |
| Human HDAC5, a.a. 656-1122 (end) | C-terminal His-tag | Baculovirus infected Sf9 cells | BPS Bioscience | | ≥ 90 % | Cat # 50005, Lot # 180115 |  |
|  |  |  |  |  |  |  |  |
| Human HDAC6, full length | N-terminal GST-tag | Baculovirus infected Sf9 cells | BPS Bioscience | | ≥ 70 % | Cat # 50006, Lot # 200410 |  |
|  |  |  |  |  |  |  |  |
| Human HDAC7, a.a. 518-952 (end) | N-terminal GST-tag | Baculovirus infected Sf9 cells | BPS Bioscience | | ≥ 90 % | Cat # 50007, Lot # 150901-G2 |  |
|  |  |  |  |  |  |  |  |
| Human HDAC8, full length | C-terminal His-tag | Baculovirus infected Sf9 cells | BPS Bioscience | | ≥ 90 % | Cat # 50008, Lot # 161216 |  |
|  |  |  |  |  |  |  |  |
| Human HDAC9, a.a. 604-1066 (end) | C-terminal His-tag | Baculovirus infected Sf9 cells | BPS Bioscience | | ≥ 76 % | Cat # 50009, Lot # 130502-20 |  |
|  |  |  |  |  |  |  |  |
| Human HDAC10, a.a. 1-481 | N-terminal GST-tag,  C-terminal His-tag | Baculovirus infected Sf9 cells | BPS Bioscience | | ≥ 23 % | Cat # 50010, Lot # 170307-A |  |
|  |  |  |  |  |  |  |  |
| Human HDAC11, full length | untagged | Baculovirus infected Sf9 cells | BPS Bioscience | | ≥ 81 % | Cat # 50021, Lot # 180122 |  |
|  |  |  |  |  |  |  |  |
| Zebrafish zHDAC6-CD2 | untagged | *E. coli* BL21 (DE3) strain | This work | | ≥ 90 % ^a^ | N/A |  |
|  |  |  |  |  |  |  |  |
| Zebrafish zHDAC6-CD2 Y745F | untagged | E. coli BL21 (DE3) strain | This work | | ≥ 90 % ^a^ | N/A |  |
|  |  |  |  |  |  |  |  |
| Zebrafish zHDAC6-CD2 H574A | untagged | E. coli BL21 (DE3) strain | This work | | ≥ 90 % ^a^ | N/A |  |
|  |  |  |  |  |  |  |  |
| Zebrafish zHDAC6-CD1 | N-terminal His/MBP-tag | E. coli BL21 (DE3) strain | This work | ≥ 90 % ^a^ | | N/A |  |
|  |  |  |  |  |  |  |  |

^a^See **Figure S7** for SDS-PAGE

**Liquid Chromatography-High Resolution Mass Spectrometry (LC-HRMS) monitored reactions of HDAC in the presence of compounds 1 and 2**

HDAC6 forms (1 µM) were incubated at 25 °C with 5 µM **1** or **2** in assay buffer, whereas HDAC3 and HDAC9 forms (1 µM) were incubated with 100 µM **1**. At different times, aliquots (40 µL) were transferred to test tubes containing acetonitrile (240 µL) to quench the reaction. Samples were kept frozen at -80 °C until LC-HRMS analysis. Quantitation of **1**, **2** and **3** in the samples was performed with respect to calibration curves obtained with varying concentrations of compounds. The calculated concentrations of compounds were plotted as percentage of compounds concentration at that time points. Alternatively, the calculated concentrations of compounds were fitted with a straight line interpolated in the linear part of the progress curve. This calculation yields a velocity value (corrected for dilutions and expressed as ΔµM/min), which was then divided by zHDAC6-CD2 concentration (µM) to calculate the apparent turnover number (min^-1^) for **1** hydrolysis.

Rapid chromatography on spin columns coupled to LC-HRMS was used to attempt the isolation of long-lived/tight complexes between HDAC6 and **1**, **2** and **3**. zHDAC6-CD2 wt (1 µM) was incubated with **1** (5 µM) or **2** (also 5 µM) as described before for 6 hours. An aliquot (60 µL) was loaded on a Bio-Spin P-6 Gel Column (Bio-Rad) that had been equilibrated with assay buffer in order to separate the HDAC6-inhibitors complexes from free compounds. After centrifugation to elute the initial fraction containing most of the enzyme, ten 200 µL aliquots were applied and eluted by centrifugation to collect the low molecular weight (free) compounds. Aliquots of the fractions were subjected to LC-HRMS for identification and quantitation of **1** and its derivatives.

The LC-HRMS analysis was carried out using a Vanquish Flex UHPLC (Thermo Fisher Scientific) and a high-resolution mass spectrometer Orbitrap QExactive Focus (Thermo Fisher Scientific), equipped with a Heated Electrospray Ionization, operated in positive mode. A Full Scan analysis was set in the m/z range 50-500 amu. A XSelect HSS T3 50x2.1 mm, 2.5 µm chromatographic column (Waters) was used. Mobile phase A consisted of 0.1% formic acid in water and mobile phase B in 0.1% formic acid in acetonitrile. The flow rate was set to 0.5 mL/min, with a gradient program from 3 to 20% B in 3 minutes.

**PROTEIN PRODUCTION AND X-RAY CRYSTALLOGRAPHY**

**Protein Production and Purification.** For recombinant protein production of the first and second catalytic domain (CD1 and CD2) of zHDAC6, the nucleotide sequences encoding residues 60-419 or 440-798 with an N-terminal TEV-cleavable 8xHis-MBP tag were codon-optimized for expression in *E. coli*, synthesized and cloned into a kanamycin-resistance expression vector (GenScript). Plasmids encoding Y745F and H574A mutants of zHDAC6-CD2 were generated by site-directed mutagenesis. The plasmids were transformed into the *E. coli* BL21 (DE3) strain, and protein expression was performed in 2xYT medium supplemented with 50 mg/mL kanamycin and 1 mM ZnSO_4_ for 30 min before induction of expression. The bacteria were cultured at 37 °C until OD_600_ of 0.2 was reached, and then the cultures were cooled to 16 °C (CD2) or 22 °C (CD1) and further incubated until OD_600_ of 1 was reached. Then, protein expression was induced with 75 µM (CD2) or 400 µM (CD1) isopropyl β-d-1-thiogalactopyranoside (IPTG), and the cultures were grown for additional 16-20 h.

The bacterial cell pellets were resuspended in buffer A (50 mM Tris pH 8.0, 500 mM NaCl, 10% (v/v) glycerol, 2 mM TCEP, 50 mM l-arginine, 50 mM l-glutamate) supplemented with 10 µg/mL DNase I and protease inhibitor cocktail and lysed by sonication. The lysate was centrifuged (18000 g, 4 °C, 1 h), and the recombinant proteins were purified from the soluble fraction using Protino Ni-NTA agarose resin (Macherey-Nagel) according to the manufacturer’s recommendations. Briefly, the soluble fraction was mixed with Ni-NTA agarose resin equilibrated with buffer A supplemented with 30 mM imidazole and incubated with gentle shaking for 1 h at 4 °C. Subsequently, the resin was washed with 30 column volumes (CVs) of buffer A supplemented with 30 mM imidazole. Next, the bound 8xHis-MBP-tagged proteins were eluted using buffer A supplemented with 300 mM imidazole. The collected proteins were subjected to size-exclusion chromatography using a HiLoad 26/600 Superdex 200 SEC column (GE Healthcare) and buffer A as the running buffer. At this point a part of 8xHis-MBP-TEV-zHDAC6-CD1 was frozen in liquid nitrogen and stored at -80 °C until use.

The fractions containing the monomeric tagged zHDAC6-CD2 proteins were pooled and treated with TEV protease (30:1 mass ratio) to remove the tag. The cleaved 8xHis-MBP tag and TEV protease were separated from zHDAC6-CD2 forms using consecutive Dextrin Sepharose (GE Healthcare) and Protino Ni-NTA affinity steps using buffer A. The target proteins were collected in the flow-through fractions and subsequently dialyzed against buffer B (50 mM HEPES pH 7.5, 100 mM KCl, 5% (v/v) glycerol, 2 mM TCEP). Finally, the proteins were polished on a HiLoad 26/600 Superdex 75 SEC column (GE Healthcare) using buffer B as the running buffer. The fractions containing the target proteins were pooled, frozen in liquid nitrogen and stored at -80 °C.

The purified proteins were analyzed by standard electrophoretic techniques (in reducing conditions) and their purities were estimated densitometrically (Gel Doc™ XR + Gel Documentation System). The SDS-PAGE analyses (**Fig. S7**) were performed according to the modified Schagger and von Jagov method, using the Tris-Tricine buffer system and 4% and 10% stacking and separating gels, respectively. The gel was run at 120 V for about 1-1.5 hours. Protein bands on the gels were visualized by staining with the CBB.

**Figure S7.** SDS-PAGE analyses of HDAC6 forms


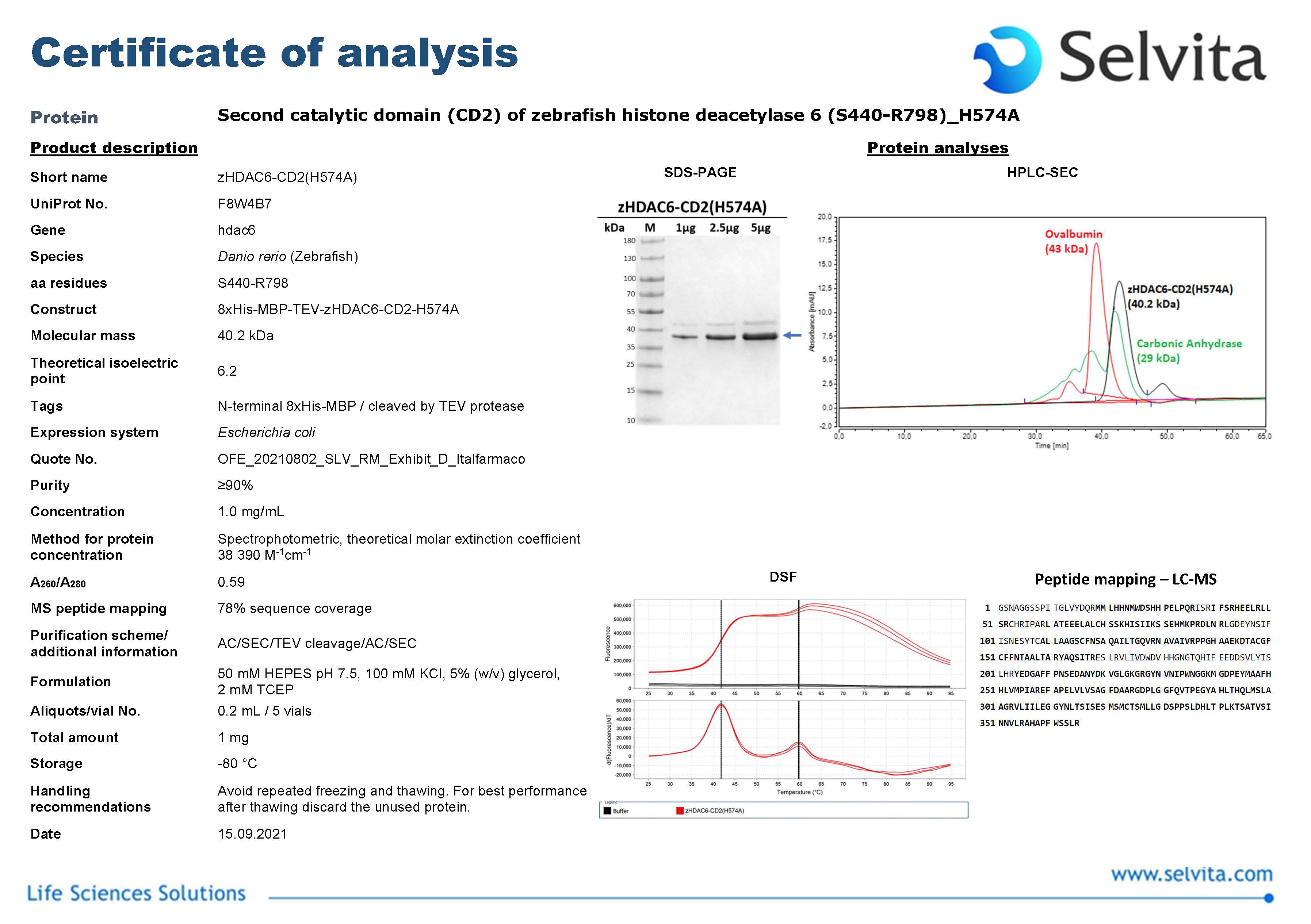

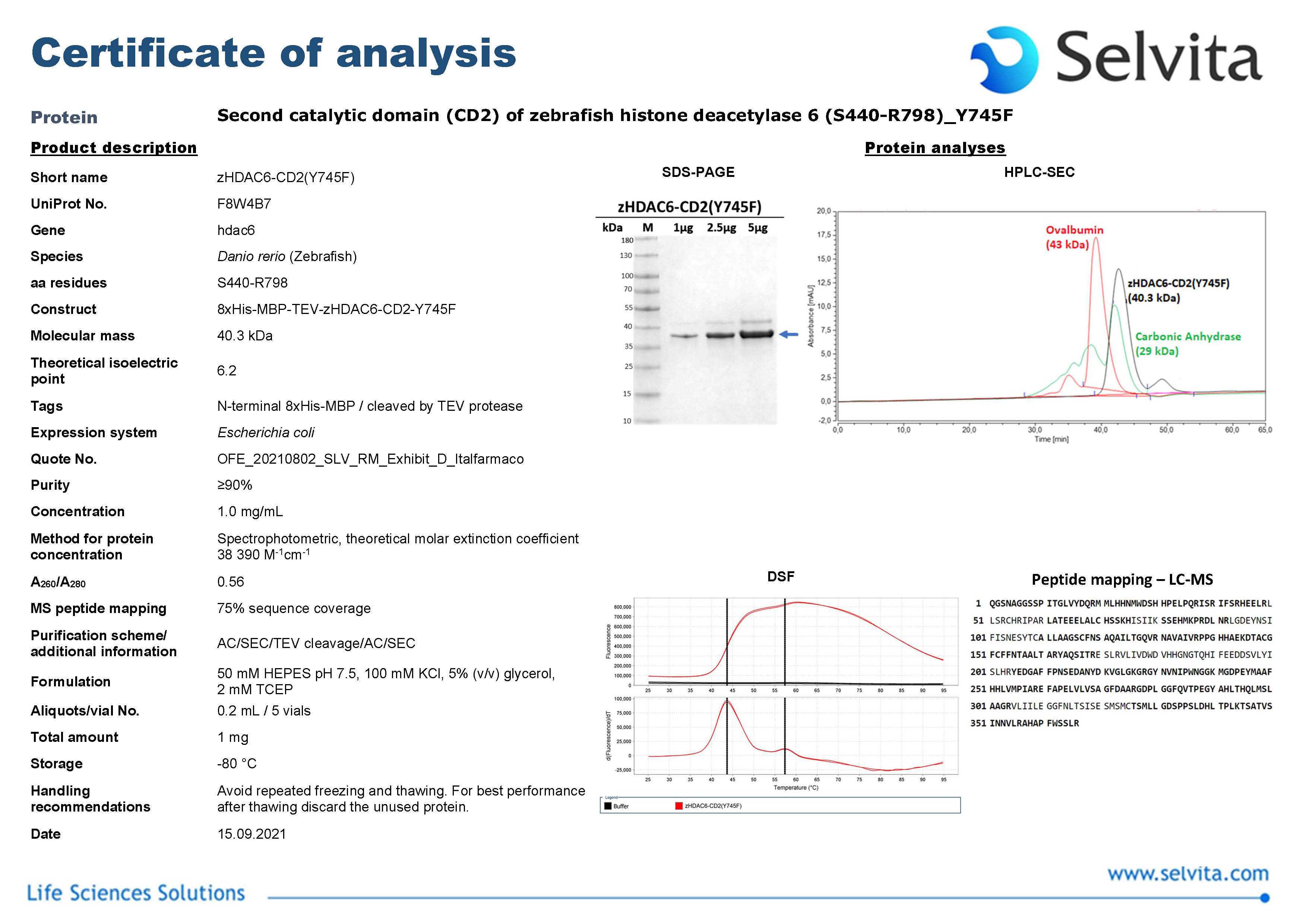

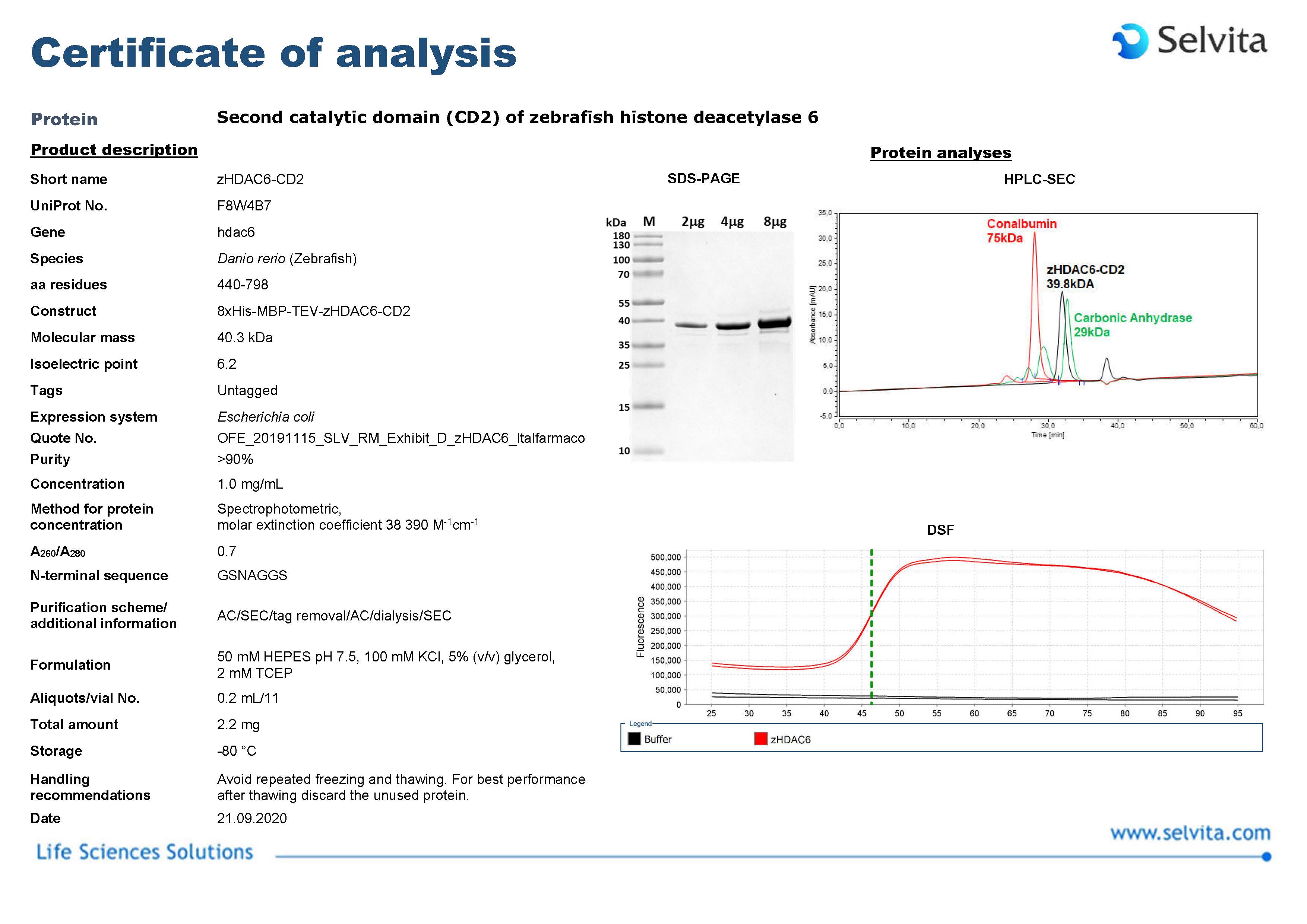


**zHDAC6-CD2 wt**

**zHDAC6-CD2 Y745F**

**zHDAC6-CD2 H574A**


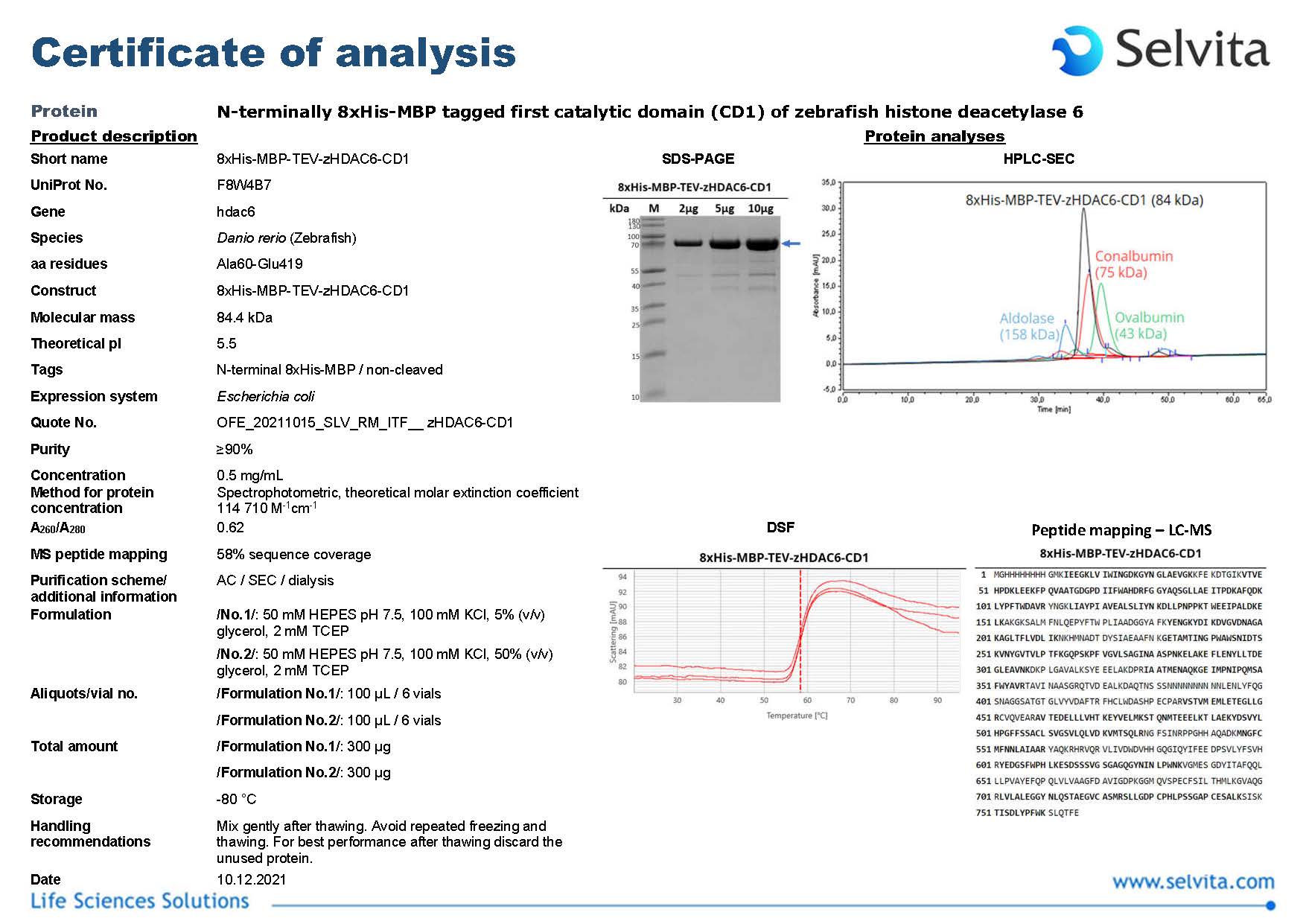


**z-His-MBP-HDAC6-CD1**


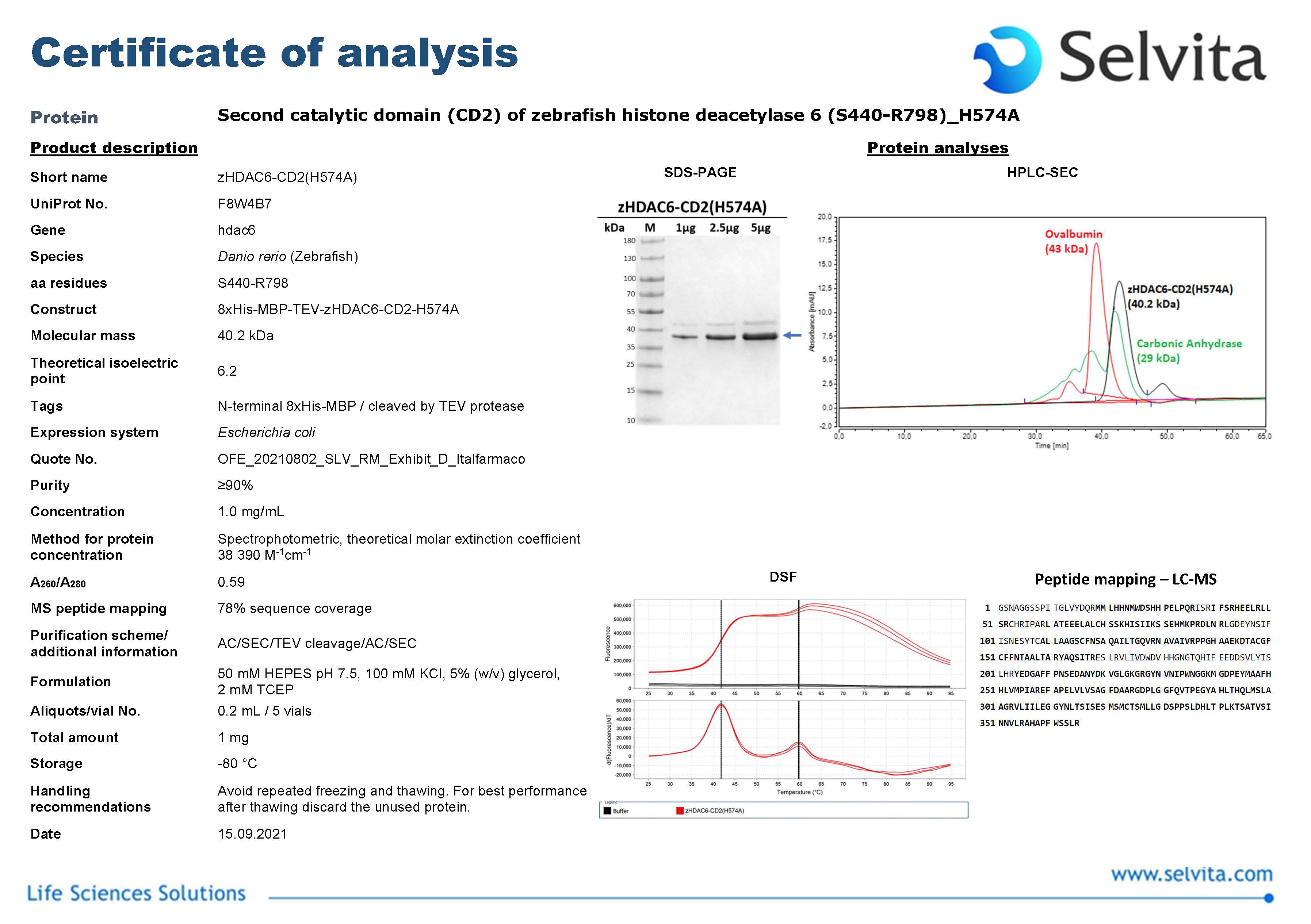


Crystallization and Data Collection. zHDAC6-CD2 at 0.2 mg/mL was mixed with 250 µM 1 (0.25% final DMSO concentration), incubated on ice for 24 h, concentrated to 10 mg/mL using a centrifugal concentration Vivaspin 20 unit (Sartorius), supplemented with additional 1 (up to 500 µM, 0.5% final DMSO concentration), incubated on ice for 2 h and subsequently used for crystallographic experiments. Screening of crystallization conditions was performed using the sitting drop vapor diffusion technique, 96-well 3-drop conical crystallization plates (Swissci), Crystal Gryphon Nanodispender (Art Robbins Instruments) and commercially available crystallization kits (Molecular Dimensions, Hampton Research, Anatrace). The prepared complex was mixed with the reservoir solution in a 1:1 drop ratio and equilibrated against 30 µL of the reservoir solution at 4 °C.

Crystals of zHDAC6-CD2-1 complex were obtained in 0.1 M Na-citrate pH 6.5, 20% (v/v) PEG4000, 20% (v/v) isopropanol and were directly flash frozen in liquid nitrogen. Data collection was performed at XRD2 beamline (ELETTRA, Trieste, Italy) at 100 K.

Data were indexed and integrated using XDS1 and scaled in Aimless from the CCP4 package (11). The crystal belonged to space groups P21 with two protein molecules per asymmetric unit.

Structure Determination. The crystal structure of the zHDAC6-CD2-ligand complex was determined by molecular replacement using Phaser (12), and the coordinates for one protein molecule of zHDAC6-CD2 from PDB entry 5EEK were used as a search model. Alternate cycles of model building and refinement were performed using WinCoot (13) and Refmac5 (14), respectively, with 5% randomly selected reflections to monitor *R_free_*. Coordinate and topology files for all ligands were generated using PRODRG (15). Details of data collection and refinement are summarized in Table S2. The atomic coordinates and crystallographic data have been deposited in the PDB under accession code 8A8Z.References

1. Lee, C. K., Ko, M. S., Yun, S. H., Lee, Y. S., and Kim, H. M. (2021) 1,3,4-oxadiazole derivative compounds as histone deacetylase 6 inhibitor, and the pharmaceutical composition comprising the same, WO2021/172887 A1

2. Kim, Y., Lee, C. S., Oh, J. T., Hyeseung, S., Choi, J., and Lee, J. (2017) Oxadiazole amine derivative compounds as histone deacetylase 6 inhibitor, and the pharmaceutical composition comprising the same, WO2017/065473 A1

3. Onishi, T., Maeda, R., Terada, M., Sato, S., Fujii, T., Ito, M., Hashikami, K., Kawamoto, T., and Tanaka, M. (2021) A novel orally active HDAC6 inhibitor T-518 shows a therapeutic potential for Alzheimer’s disease and tauopathy in mice. *Sci. Rep.* 10.1038/s41598-021-94923-w

4. Glide Schrödinger, LLC, New York, NY, 2021

5. LigPrep Schrödinger, LLC, New York, NY, 2021

6. Epik Schrödinger, LLC, New York, NY, 2021

7. QSite Schrödinger, LLC, New York, NY, 2021

8. Hai, Y., and Christianson, D. W. (2016) Histone deacetylase 6 structure and molecular basis of catalysis and inhibition. *Nat. Chem. Biol.* **12**, 741–747

9. Copeland, R. A. (2013) *Evaluation of Enzyme Inhibitors in Drug Discovery*, Wiley, 10.1002/9781118540398

10. Rubinson, K. A. (2017) Practical corrections for p(H,D) measurements in mixed H2O/D2O biological buffers. *Anal. Methods*. **9**, 2744–2750

11. Winn, M. D., Ballard, C. C., Cowtan, K. D., Dodson, E. J., Emsley, P., Evans, P. R., Keegan, R. M., Krissinel, E. B., Leslie, A. G. W., McCoy, A., McNicholas, S. J., Murshudov, G. N., Pannu, N. S., Potterton, E. A., Powell, H. R., Read, R. J., Vagin, A., and Wilson, K. S. (2011) Overview of the CCP4 suite and current developments. *Acta Crystallogr. Sect. D Biol. Crystallogr.* **67**, 235–242

12. McCoy, A. J., Grosse-Kunstleve, R. W., Adams, P. D., Winn, M. D., Storoni, L. C., and Read, R. J. (2007) Phaser crystallographic software. *J. Appl. Crystallogr.* **40**, 658–674

13. Emsley, P., Lohkamp, B., Scott, W. G., and Cowtan, K. (2010) Features and development of Coot. *Acta Crystallogr. Sect. D Biol. Crystallogr.* **66**, 486–501

14. Murshudov, G. N., Skubák, P., Lebedev, A. A., Pannu, N. S., Steiner, R. A., Nicholls, R. A., Winn, M. D., Long, F., and Vagin, A. A. (2011) REFMAC5 for the refinement of macromolecular crystal structures. *Acta Crystallogr. Sect. D Biol. Crystallogr.* **67**, 355–367

15. Schüttelkopf, A. W., and Van Aalten, D. M. F. (2004) PRODRG: A tool for high-throughput crystallography of protein-ligand complexes. *Acta Crystallogr. Sect. D Biol. Crystallogr.* **60**, 1355–1363
